# A locus coeruleus-centric framework for novelty, salience, and gamma oscillations: implications for neurostimulation, aging, and disease

**DOI:** 10.3389/fnagi.2026.1895931

**Published:** 2026-08-03

**Authors:** Brennan L. Jackson

**Affiliations:** Department of Brain and Cognitive Sciences, Massachusetts Institute of Technology, Boston, MA, United States

**Keywords:** aging, Alzheimer’s disease and dementia, gamma oscillations, locus coeruleus (LC), noradrenergic signaling, novelty and encoding

## Abstract

Neuromodulation techniques in the gamma frequency range, such as chronic 40 Hz sensory stimulation, have gained attention for their potential to impact and preserve cognitive function in the context of neurodegenerative disorders such as Alzheimer’s disease (AD). This hypothesis and theory article aims to examine the overlap in biological phenotypes between gamma stimulation techniques and to propose a theoretical mechanism of action that may help to inform future empirical investigations. In Part 1, we provide a neuromodulatory framework related to a multifaceted novelty/salience circuit consisting of phasic activation of hind-, mid-, and basal forebrain nuclei, especially the locus coeruleus (LC); the release of norepinephrine (NE); the activation of cholinergic, dopaminergic, and serotonergic nuclei; and downstream modulation of the neurogliavascular unit through astrocyte-dependent pathways in order to reduce neuroinflammation, increase blood and glymphatic flow, promote synaptic plasticity and myelination, and improve hippocampal-dependent memory. We suggest various falsifiable experiments and testable modifications to stimulation paradigms based on this hypothesis. In Part 2, we review and explore the role of these hind- and midbrain nuclei and neuromodulators in terms of learning, allostatic load, consciousness, and sleep. To strengthen the initial hypothesis and highlight the significance of this proposed circuit, we cite observations of altered gamma oscillations across a variety of neuropsychiatric disorders, as well as speculate on the role of brainstem nuclei in current and potential treatments. This portion aims to emphasize the critical importance of the neural circuit proposed, its involvement in conscious experience, and to potentially inform future neuropsychiatric research and disease modeling, as well as indications for LC-NE or gamma frequency-based interventions. Part 3, in order to explain the significant impact of 40 Hz stimulation on Alzheimer’s disease and neurodegeneration, explores a hypothetical functional framework for the core role of the LC-NE system in novelty, evolutionary drive, and management of allostatic load, its potential role in vertebrate aging, and a hypothesis for LC-NE dysfunction and depletion as a significant upstream modulator of the development of amyloid plaques and Alzheimer’s disease, respectively. The goal of this work is to motivate future empirical investigations into 40 Hz stimulation paradigms and biomarkers proposed, studies assessing the role of the LC-NE system in the conduction of consciousness and alterations in neuropsychiatric disorders discussed, and into the potential role of the LC in vertebrate aging and the development of Alzheimer’s disease.

## Graphical Abstract

**Figure:**
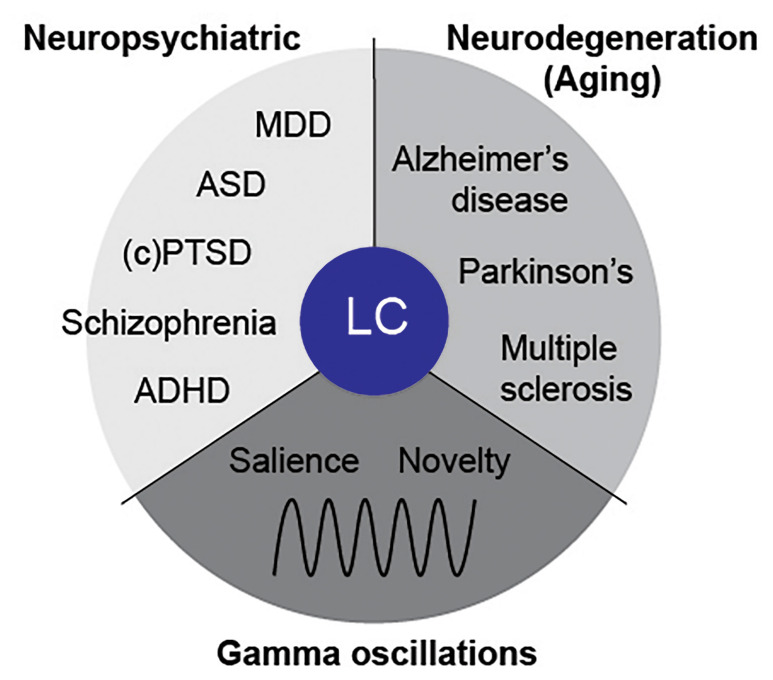
Illustrates the proposed mechanistic framework linking phasic locus coeruleus (LC) activation with novelty, salience, and gamma oscillations. The manuscript reviews data and explores implications for neurostimulation in the gamma frequency range, sleep, and aging, as well as neuropsychiatric and neurodegenerative disorders. ADHD, attention-deficit/hyperactivity disorder; ASD, autism spectrum disorder; (c)PTSD, (complex) post-traumatic stress disorder; MDD, major depressive disorder.

## 1 A potential role for novelty and attention and noradrenergic and cholinergic signaling in gamma frequency stimulation

### 1.1 Introduction

**FIGURE 1:**
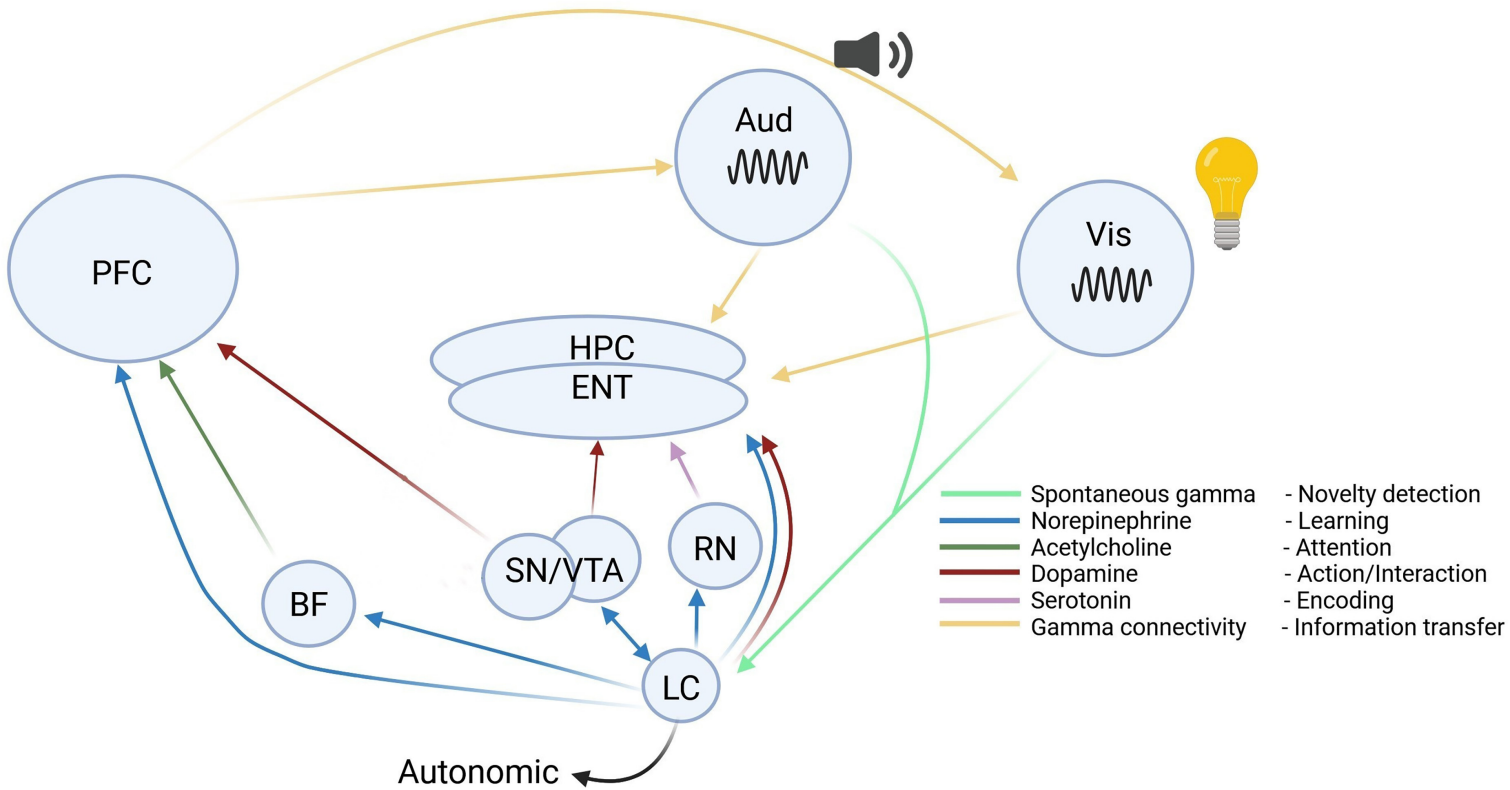
Novelty, salience, and selective attention circuit. Spontaneous gamma oscillations occur in the sensory cortex in response to unexpected, novel, or salient stimuli. Hind-, mid-, and forebrain response promotes attention, engagement, and interaction, using cholinergic, dopaminergic, and noradrenergic signaling to recruit neural circuits and metabolic resources. Increased prefrontal activity promotes sensory-hippocampal connectivity and memory encoding. LC, locus coeruleus; BF, basal forebrain; Aud, auditory cortex; Vis, visual cortex; PFC, prefrontal cortex; HPC, hippocampus; ENT, entorhinal; RN, raphe nucleus; SN, substantia nigra; VTA, ventral tegmental area.

Gamma oscillations within the sensory cortices, consisting of the synchronization of neural groups in the 30–100 Hz band, have been shown to play a key role in feature binding, multisensory information encoding, selective attention, and increased information transmission to the hippocampus (Guan et al., 2022). The process of the cortex promoting arousal, attention, and determining novelty is thought to rely on a complex interplay of dopaminergic and noradrenergic signaling for contextual novelty and cholinergic and noradrenergic signaling encoding absolute novelty (Kafkas and Montaldi, 2018). Below, we review the significant impacts of Gamma ENtrainment Using Sensory Stimulation (GENUS) on (1) glial cells and neuroinflammation, (2) increasing blood and glymphatic flow, (3) plasticity, neurogenesis, and preventing neurodegeneration, and (4) hippocampal dependent memory in preclinical models of Alzheimer’s disease (AD) and neurodegeneration, as well as related phenotypes observed in a variety of disease models including chemobrain, multiple sclerosis, Down syndrome, and stroke. Additionally, similar results observed using vagus nerve and transcranial stimulation in the gamma range are discussed. We propose a potential mechanism in which the positive impact of GENUS and gamma-range stimulation may be mediated via phasic activation of hind- and basal forebrain nuclei, promotion of noradrenergic and cholinergic signaling, influencing of glial and synaptic dynamics, and promotion of circuit health (Figure 1). We hypothesize that these phenotypes may depend upon neuromodulatory communication in addition to direct neuron-glia interactions within the cortex and corticocortical communication. We discuss the ways in which this circuit has been shown to be functionally impacted by the pathophysiology of AD and its relationship to cognitive reserve and decline. Finally, to support future research in these directions, we discuss several falsifiable experiments and potential improvements in stimulation protocols to more directly promote this signaling pathway.

### 1.2 Gamma oscillations in sensory processing and encoding

Gamma oscillations in the cortex, characterized by rapid excitation and decreased inhibition, have been shown to play a fundamental role in network information flow and communication during attention, cognitive processing, and memory encoding and retrieval (Paul et al., 2004). Selective attention, the process of filtering salient stimuli from irrelevant stimuli, is thought to involve gamma rhythmic coordination of cells responding to salient stimuli (Fries, 2015).

Sensory stimulation and behavioral responses dynamically modulate the functional connectivity between pyramidal cells and inhibitory interneurons, including parvalbumin (PV) expressing, somatostatin expressing, and vasoactive intestinal peptide (VIP) interneurons (Antonoudiou et al., 2020; Dipoppa et al., 2018). Synaptic connections between inhibitory interneurons, particularly PV, and their associated excitatory neurons mediate the short-term plasticity of synaptic inhibition and the consequent endogenous gamma oscillations. The prevailing understanding is that the inhibitory interneuron network characterized by these unique discharges and synaptic activities (referred to as the Pyramidal Interneuron Network Gamma, or PING) is essential for the generation of gamma rhythm (Buzsáki and Wang, 2012; Cardin, 2018).

In addition to inhibitory Gamma-Aminobutyric Acid-(GABA)ergic transmission, a neuronal network with asynchronous firing can produce gamma oscillations in response to acetylcholine (ACh) pulses via muscarinic ACh receptors (mAChRs), which enables the dynamic network adaptation during attentional tasks (Lu et al., 2020). Cue detection in behavioral attention tasks is dependent on cholinergic-driven gamma oscillations in the frontal cortex, in which both mAChRs and nicotinic ACh receptors (nAChRs) make distinct contributions (Howe et al., 2017). Recent experimental results have shown that the detection of cues in behavioral attention tasks relies on transient increases of acetylcholine release in frontal cortex and cholinergically driven oscillatory activity in the gamma frequency band. Norepinephrine (NE, or noradrenaline) input also modulates rhythmic neuronal activity during selective attention and bidirectionally influences hippocampal gamma activity (Dahl et al., 2022; Haggerty et al., 2013).

Cortical acetylcholine and norepinephrine input are largely mediated by the basal forebrain (BF) and locus coeruleus (LC), respectively. In the adult mammalian brain, cholinergic projection neurons of the basal forebrain span the entire extent of the cortex (Ananth et al., 2023). Cholinergic neurons participate in essential functions including respiration, sleep, attention, mood, and memory and act via two major classes of receptors: G protein-coupled mAChRs and ionotropic nAChRs. The LC is a cluster of relatively large neurons containing NE that is located bilaterally in the brainstem just under the cerebellum and lateral to the fourth ventricle (Poe et al., 2020). The distribution of LC-NE axonal fibers is nonuniform across the rat and primate neocortex and coordinated to target functionally related circuits, such as those coordinating aversion and/or anxiety in the anterior cingulate and amygdala. The LC extensively innervates the hippocampus and LC release of both norepinephrine and dopamine plays a critical role in hippocampal memory encoding (Weinshenker, 2018). Additional evidence for projections from a subgroup of LC neurons to the caudate–putamen, along with increased NE turnover in striatum and the functional connectivity of striatal–motor networks, is revising previous conceptions that LC-NE projections do not influence striatal–motor function (Zerbi et al., 2019). The projection of the LC to other motor structures such as the cerebellum has not been fully characterized.

Overall, data indicate that interactions between the hindbrain nuclei and anterior hippocampus play a crucial role in encoding salience and novelty of a given stimulus (Kafkas and Montaldi, 2018). In response to novelty, noradrenergic input to the hippocampus (HPC) increases gamma oscillations and promotes plasticity and encoding while also increasing information transmission through the perforant path. Fast gamma rhythms (>65 Hz) in the hippocampus are coupled with fast gamma inputs from the medial entorhinal cortex responsible for processing current sensory information, while slow gamma rhythms (30–50 Hz) show coherence within hippocampal regions (Colgin et al., 2009). It has been proposed that fast gamma promotes the transmission of current sensory information to the hippocampus during memory encoding (Colgin, 2016). Fast gamma power dominates within the hippocampus during novel object location tasks (Zheng et al., 2016). Cholinergic signaling significantly modulates hippocampal gamma oscillations and theta-gamma coupling (Betterton et al., 2017; Fisahn et al., 1998; Yang et al., 2021). In the setting of multisensory input and crosstalk, it has been shown that corticocortical synchronized gamma oscillations are induced in order to modulate information flow during a visual-tactile stimuli task and that circuit modulation using transcranial alternating current stimulation at 40 Hz can impact these relationships (Mably and Colgin, 2018).

Outside of promotion and involvement in memory encoding in response to salient and/or novel sensory stimuli, slow gamma power in the hippocampus paired to sharp-wave ripples (SWRs) during rest and slow wave sleep may support a functional role in memory retrieval (Misselhorn et al., 2019).

In summary, gamma oscillations within the sensory cortices can arise as a result of spontaneous endogenous production in response to a salient stimulus based on asynchronous inhibitory activity. In the setting of hind-, mid-, and forebrain phasic activation, these oscillations may be sustained as a result of cholinergic input from the basal forebrain driving gamma oscillations in the prefrontal cortex promoting sustained attention. Simultaneously, gamma oscillations in the hippocampus result from noradrenergic, cholinergic, serotonergic, or downstream brain-derived neurotrophic factor (BDNF) input to promote memory encoding of novel stimulus or experience (Huang and Morozov, 2011). LC activation facilitates sensory processing, enhances cognitive flexibility and executive function in the frontal cortex, and prepares the organism for reorientation or adaptation (Sara and Bouret, 2012). NE is a key neuromodulator for the regulation of behavioral state and cognition; supporting learning by increasing arousal and vigilance to “earmark” new experiences for encoding (Hagena et al., 2016).

In total, sensory stimuli evoke spontaneous gamma oscillations, prompting the determination of the novelty and saliency of the stimulus, and may activate noradrenergic and cholinergic signaling to produce sustained gamma oscillations in the sensory, prefrontal, and hippocampal cortices, as well as increased transmission along the perforant path. This response has been well-characterized.

### 1.3 Gamma ENtrainment Using Sensory Stimulation (GENUS)

Gamma ENtrainment Using Sensory Stimulation, which exposes users to light, sound, or vibratory stimuli modulated at 40 Hz for 1 h/day for a period of weeks, has shown success in ameliorating amyloid and tau pathology, reducing neuroinflammation and neurodegeneration, and reducing cognitive deficits and decline in various mouse models of AD and human studies (Blanco-Duque et al., 2024). Impacts on circadian genes and sleep structure have been observed in mouse models and subjects with mild AD, respectively (Chan et al., 2022; Yao et al., 2020). Significant changes in glial cell populations, immune profiles, and cerebral spinal fluid (CSF) proteomics across multiple cohorts have been reported (He et al., 2021). Many of these benefits are thought to be associated with increased glymphatic clearance during and following 40 Hz stimulation as a result of VIP+ interneuron activity and adenosine signaling (Murdock et al., 2024; Sun et al., 2024). Chronic GENUS resulted in reduced α-synuclein deposition and depressive behaviors and improved neuromuscular strength and spatial working memory in a mouse model of Parkinson’s disease (PD) (Liu et al., 2023). 12-weeks of physioacoustic 40 Hz stimulation found improved tremor, rigidity, bradykinesia, and posture and gait measures in patients with PD (Mosabbir et al., 2020). Additionally, GENUS has also been found to promote benefits in stroke and ischemia models, reduce demyelination in multiple sclerosis, myelination and cognitive function in chemobrain, and increase neurogenesis, cognitive function, and hippocampal synapse number in a mouse model of Down syndrome (Islam et al., 2025; Kim et al., 2024; Niu et al., 2023; Rodrigues-Amorim et al., 2024). Unlike previous studies, Soula et al. (2023) did not observe entrainment of gamma oscillations or impact on amyloid clearance, but found increased hippocampal cholinergic tone. Yan et al. (2025) found significant 40 Hz entrainment in the hippocampus, no change in cholinergic activity in the dentate gyrus, and significantly increased neurogenesis. For a summary of preclinical and clinical results of chronic sensory 40 Hz stimulation, see Table 1. We note that the works cited here include large variation in disease model and mechanisms, stimulation protocols and modalities used, and outcome measures reported, and we have attempted to broadly summarize some of those outcomes into the categories below.

**TABLE 1:** Preclinical and clinical impacts of 40 Hz sensory stimulation.

Publication	Biological Phenotypes							Functional and cognitive phenotypes				
	Synapse structure or function	Neuroinflammation or glial involvement	Neuron number or brain structure	White matter	Vascular	Glymphatic	Network function	Sleep	Behavior or function		Motor	Memory
Alzheimer’s disease												
Iaccarino et al., 2016	X		X									
Adaikkan et al., 2019		X	X	X								X
Martorell et al., 2019	X		X									X
Yao et al., 2020	X								X			
Park et al., 2020	X	X	X									X
Park et al., 2022	X	X	X									X
Shen et al., 2022	X											
Yang et al., 2023		X	X									
Suk et al., 2023	X	X		X								X
Murdock et al., 2024						X	X					
Soleimani et al., 2024										X		
Paulson et al., 2024								X				X
Barzegar Behrooz et al., 2024		X										X
Wu et al., 2025							X			X		X
Yang et al., 2025	X											
He et al., 2021			X					X				
Cimenser et al., 2021									X	X		
Chan et al., 2022				X				X	X	X		
Hajós et al., 2024				X						X		X
Parkinson’s disease												
Liu et al., 2023	X			X						X	X	X
Mosabbir et al., 2020											X	
Down syndrome												
Islam et al., 2025		X		X								X
Stroke or brain injury												
Zheng et al., 2020		X						X			X	X
Zhu et al., 2022		X	X							X		
Niu et al., 2023		X	X									X
Yang et al., 2022								X			X	
Wang et al., 2023		X						X				X
Neuropsychiatric												
Ju et al., 2024										X		
Braun Janzen et al., 2019*									X	X		
Multiple sclerosis												
Rodrigues-Amorim et al., 2024		X	X		X							
Sleep deprivation or stress												
Wang et al., 2024								X		X		X
Zhou et al., 2024		X							X			
Yao et al., 2024								X		X		X
Chemobrain												
Kim et al., 2024	X	X		X	X							X
Clinical study	*30–70 Hz stimulation											

Outside of sensory stimulation, 40 Hz stimulation using transcranial magnetic stimulation (TMS) of the angular gyrus was found to reduce volume loss in a variety of regions in patients with probable AD (Liu et al., 2022). 40 Hz transcranial alternating current stimulation (tACS) of the temporal lobe was found to increase perfusion and improve cognition in patients with mild to moderate AD (Sprugnoli et al., 2021). Transauricular vagus nerve stimulation (taVNS) at 40 Hz found reduced pathology, modulated microglia, and improved spatial memory in a mouse model of AD (Yu et al., 2023). The vagus nerve is often viewed as a stimulus reporter from “other” senses (Prescott and Liberles, 2022). Proprioception, satiety, peripheral inflammation, and other inputs all make up important contextual information that the hind- and midbrain nuclei and anterior hippocampus need to determine novelty and salience when deciding whether to make behavioral changes in response to an experience or input. We hypothesize that gamma stimulation of vagus nerve branches may induce similar salience/novelty activation to circuits outside the central nervous system; resulting in previously documented cholinergic and noradrenergic output to relevant regions (Berger et al., 2024).

**FIGURE 2:**
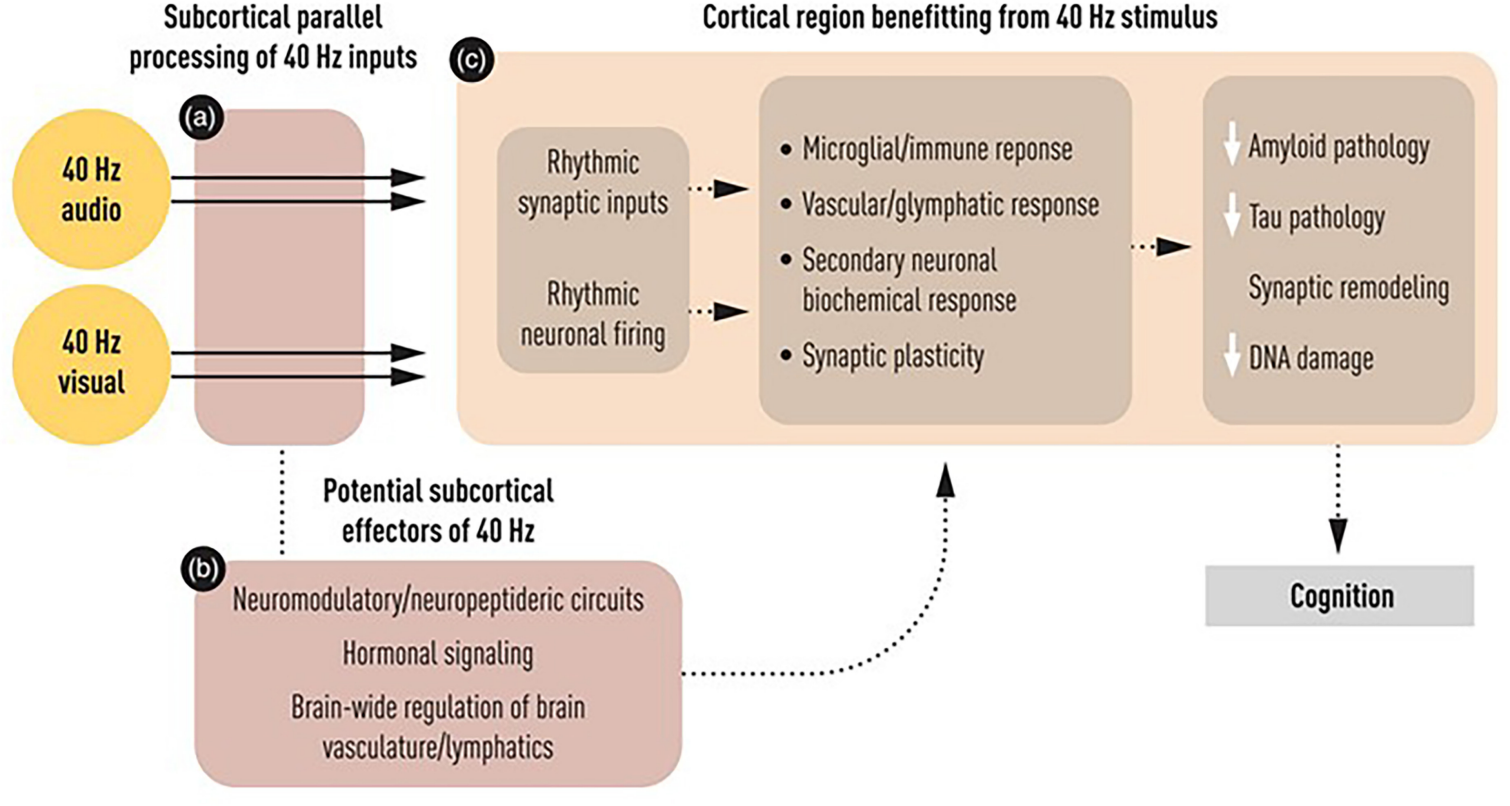
Mechanisms underlying the beneficial effects of gamma stimulation in AD schematic showing known and hypothetical mechanisms mediating the clinical effects of 40 Hz stimulation. From Blanco-Duque et al. (2024) **(a)** sensory stimulation is transduced in the sensory organs and the signals progress through the canonical sensory pathways. Note that sensory processing is highly parallelized (indicated by parallel black arrows); hence, 40-Hz stimulation not only is relayed to cortex via thalamus but also travels via parallel pathways to multiple subcortical regions and can influence other modalities via regions such as the thalamic reticular nucleus. **(b)** Hypothetical involvement of subcortical circuits that process sensory stimulation in parallel to cortical signaling. **(c)** A region that responds to 40 Hz stimulation (e.g., amyloid reduction and neuroprotection) may do so due to direct subthreshold synaptic currents (e.g., evoking hemodynamic changes or synaptic plasticity, changes in neuronal firing) or due to nonlocal events (e.g., global increase in CSF flow and neuromodulators). CSF, cerebrospinal fluid.

Overall, across these disease models and stimulation protocols, the proposed impact of GENUS could be summarized similar to Blanco-Duque (Figure 2) as (1) changes in glial cells and neuroinflammation (2) increases in blood and glymphatic flow (3) increases in plasticity and neurogenesis within the hippocampus, and as a result, improvements in hippocampal-dependent memory (Blanco-Duque et al., 2024).

Hypothesis: We propose that inducing gamma oscillations in multiple sensory cortices mimics activation of the novelty/salience circuit described above by inducing similar activation patterns and corresponding noradrenergic and cholinergic input. Doing this chronically may mimic day-to-day strong sustained novelty/learning-inducing experiences involving the activated neural pathways and sensory cortices. In other words, we propose that driving sensory cortical firing at 40 Hz mimics both the initial endogenous gamma activity produced by inhibitory/excitatory neuron firing and sustained encoding of novel or salient sensory stimuli processing due to LC-NE activation and prefrontal cholinergic input. We hypothesize that this would feed back into the circuit to promote sustained attention and novelty encoding through noradrenergic and cholinergic activation. We would then hypothesize that a portion of therapeutic benefits of GENUS may be due to phasic activation of brainstem nuclei and increased neuromodulatory tone. Other changes or benefits of GENUS may result from mechanisms unrelated to LC activity.

In the following sections, we outline how each of the above categories of 40 Hz stimulation impact may be a result of activation of this salience and novelty circuit producing an increase in cholinergic and noradrenergic output to the sensory, prefrontal, and hippocampal cortices.

#### 1.3.1 Glial cells and neuroinflammation

Several studies indicate that microglia and astrocytes express adrenergic receptors, whose activation influences the release of pro-inflammatory mediators, controlling the extent of glial reactivity (Sugama et al., 2019). Acetylcholine receptors are also expressed by glial cells (Gamage et al., 2020). Microglial cells express the nicotinic receptor α7 and its activation attenuates the pro-inflammatory response of microglial cultures, suggesting that acetylcholine may play a role in controlling brain inflammation, analogous to what is demonstrated in peripheral tissues (Pavlov and Tracey, 2006). This highlights the role that noradrenergic and cholinergic input have on both overall central nervous immune system reactivity and immediate inflammatory response, respectively.

**FIGURE 3:**
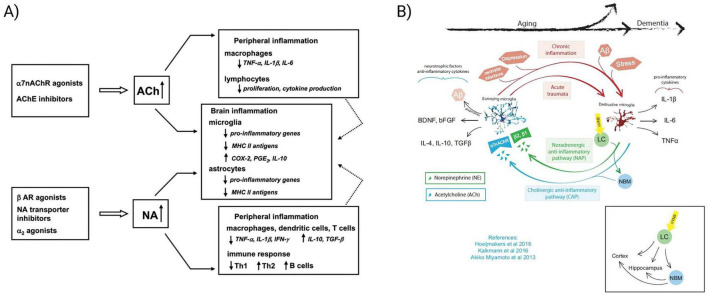
Impact of noradrenaline and acetylcholine signaling on peripheral and central immune systems. **(A)** From Carnevale et al. (2007) schematic representation of some of the putative strategies to promote the cholinergic (ACh) and noradrenergic (NA) anti-inflammatory pathways. Through their activities on cells of the immune system and on glial cells (microglia and astrocytes), the two neurotransmitters may control inflammation in both peripheral and brain inflammatory processes. By controlling inflammation in the periphery, ACh and NA could also prevent the positive feed-back of systemic inflammation on brain inflammation and neurodegeneration. **(B)** From Kaczmarczyk et al. (2018) interaction scheme between microglia and non-invasive vagus nerve stimulation (nVNS). Stimulation of the locus coeruleus (LC) and the Nucleus Basalis of Meynert (NBM) following nVNS leads to activation of the cholinergic (CAP) and the noradrenergic (NAP) anti-inflammatory pathways. The LC fibers lead widely throughout the brain to areas like the NBM, Hippocampus and the cerebral cortex. The most prevalent neurotransmitters here are NA and ACh. The suggested target on microglia cells for ACh is the a7nAChR, whereas NA mainly activates b1 and b2 receptors. The activation of these pathways is associated with a general anti-inflammatory response. Healthy microglia have better Aβ phagocytosis capabilities and increased release of neurotrophic factors (BDNF, bFGF), and anti-inflammatory cytokines (IL-4, IL-10, TNFb). These surveying microglia (represented on the left side of the figure in blue) which typically show long, ramified branches also help pruning of neuronal synapses. During aging, and even more in addition to external (infections, acute traumata) or internal (Ab deposition, stress, depression) stimuli, microglia cells get primed or even chronically activated. This leads to neuroinflammation and a toxic environment in which pro-inflammatory cytokines (IL1-b, IL-6, TNFa) of the now amoeboid cells with retracted processes get released (represented on the right side of the figure in red). This proinflammatory environment can lead to extensive neuronal loss and in the end dementia.

The cholinergic anti-inflammatory pathway (CaiP) has been well-documented and studied (Han et al., 2017; Martelli et al., 2014; Pavlov and Tracey, 2006). NE inhibits microglial activation and suppresses pro-inflammatory mediator production (e.g., tumor necrosis factor-α, interleukin-1β and inducible nitric oxide synthase activity), thus limiting the cytotoxicity of midbrain dopaminergic neurons in response to an inflammatory stimulus (O’Neill and Harkin, 2018). The anti-inflammatory effect of vagal stimulation depends on the presence of noradrenaline-releasing nerve terminals in the spleen, as well as a variety of neuroplasticity and anti-inflammatory vagus nerve stimulation (VNS) outputs being directly related to the CaiP (Berger et al., 2024; Mota and Madden, 2022). Common immune biomarkers of noradrenergic anti-inflammatory pathway (NaiP) and CaiP include TNF-α, IL-1β, IL-6, IL-10, and TGF-β (Figure 3; Carnevale et al., 2007; Kaczmarczyk et al., 2018).

Neuroinflammation and oxidative stress driven by microglia can disrupt normal gamma oscillations. Minocycline, a microglia inhibitor, alleviates neuroinflammation and synaptic loss, which consequently restores gamma oscillations in the prefrontal circuit and improves cognitive deficits in a dual genetic-environmental mouse model of neuropsychiatric disorder (Chini et al., 2020; Ji et al., 2020).

Astrocytes are responsive to a range of neuromodulators. NE evokes robust calcium transients in astrocytes across brain regions through activation of α1-adrenoreceptors (Wahis and Holt, 2021). Astrocytes ensheathe neurons at synapses and are known to modulate synaptic activity. Hence, astrocytes are in a key position to relay, or amplify, the effects of NE on neurons, most notably by modulating inhibitory transmission. Cortical NE-astrocyte interactions have been shown to be critical to learning behavior (Drummond et al., 2024). Previous data suggest a role of cholinergic precursors on maturation and differentiation of astroglial cells *in vitro* (Bramanti et al., 2008).

Using cholinergic agonists in hippocampal slices, Lee et al. (2014) investigated gamma oscillations and found that the transient increase of calcium concentration in astrocytes precedes the onset of oscillations and that the release of astrocyte vesicles is necessary for the maintenance (but not the initiation) of cholinergic-induced gamma oscillations, as well as normal cognition and memory in animals. Astrocyte specific S100 calcium binding protein B (S100B) in the medial prefrontal cortex (mPFC) enhances theta-gamma phase-amplitude coupling *in vivo* and improves cognitive flexibility, indicating that astrocytes might participate in the complicated signaling that constitutes the neural circuits of advanced cognitive function (Brockett et al., 2018). Furthermore, PV+ interneurons in mPFC recruit astrocytes to support the generation of gamma oscillations and to rectify decision-making behavior, as astrocyte regulation of signal transmission is critical to the maintenance of gamma oscillations (Guan et al., 2022; Makovkin et al., 2022).

Noradrenergic input also modulates calcium signaling and promotes oligodendrocyte precursor cell differentiation during exploratory behavior (Fiore et al., 2022). Adrenergic signaling to astrocytes and oligodendrocytes provides critical support to maintenance of white matter integrity and may play a role in demyelination observed in multiple sclerosis (De Keyser et al., 2004; Papanikolaou and Butt, 2017). Choline signaling is critical to oligodendrocyte differentiation and myelin sheath formation in the postnatal brain (Fields et al., 2017; Liu et al., 2025). Myelinating glia express all different cholinergic receptors at different periods of development and have high expression of cholinesterases.

Overall, microglia, astrocytes, and oligodendrocytes, as well as various progenitors, have been shown to extensively interact with cholinergic and noradrenergic inputs and gamma oscillations and that these inputs not only impact immediate glial state, but also glial proliferation and propensity toward a chronic inflammatory state. Neuromodulator-glial interactions have been shown to have a profound impact on circuit function and oscillatory dynamics through interactions across the neurogliavascular unit and impact on inhibitory transmission during learning behavior, discussed more below. Given the hypothesized increase in noradrenergic and cholinergic input following GENUS, we would expect significant changes in inflammatory tone and microglial surveillance, as well as astrocyte maturation and differentiation. These phenotypes have been consistently observed across disease models after chronic exposure to GENUS (Adaikkan et al., 2019; Iaccarino et al., 2016; Martorell et al., 2019).

#### 1.3.2 Blood and glymphatic flow to relevant circuits

The intracerebral cholinergic fibers projecting from the basal forebrain are able to act as autonomic nerve fibers for the regulation of cortical and hippocampal blood flow (Sato et al., 2004). Activation of these cholinergic vasodilator fibers results in the release of ACh within the cortex, activation of both nicotinic and muscarinic ACh receptors, and vasodilation without coupling to glucose metabolic rates. This cholinergic vasodilator system has been shown to decline with age in rats mainly due to age-related declines of nicotinic ACh receptor activity (Hotta et al., 2011). Choline and its downstream products such as choline-containing phospholipid particles have also been shown to play a critical role in multiple components of the neurogliavascular unit including neurons, astrocytes, microglia, and endothelial cells themselves (Roy et al., 2022).

At the local neuronal level, NE suppresses most activity but amplifies the strongest activity as a result of the differential effects on adrenoreceptor subtypes (Mather et al., 2016). The amplification of strong activity occurs via “NE hotspots,” where positive feedback loops between local NE and glutamate release increase the strength of activated representations. To sustain higher levels of activity, hotspots also recruit additional metabolic resources. At the circuit level, the increased glutamate and NE produced at hotspots recruit nearby astrocytes that supply energy to active neurons. On a broader scale, NE facilitates the redistribution of blood flow toward hotspots and away from areas of lower activity (Mather et al., 2016).

Norepinephrine has also been shown to be a key regulator of glymphatic activity and its suppression during wakefulness (Jessen et al., 2015). Application of NE or antagonists result in inverse CSF-interstitial fluid (ISF) exchange and CSF production. Rhythmic norepinephrine oscillations also regulate the slow vasomotion patterns necessary for glymphatic clearance during non-rapid eye movement (NREM) sleep (Plog et al., 2025). Work from Maiken Nedergaard recently showed that in the setting of traumatic brain injury, noradrenergic input uniquely influences central glymphatic flow (Hussain and Nedergaard, 2024).

Hypothesis: We propose that the mechanism of noradrenergic and cholinergic control over central glymphatic flow is a combinatorial influence on astrocytes, GABAergic interneurons, and the interaction between the two neuromodulators influencing the neurogliavascular unit and autonomic tone. Additionally, the increased blood flow, vasomotion, and glymphatic clearance observed following GENUS may be partially mediated via neuromodulator influence. Changes in adenosine signaling following GENUS observed by Sun et al. (2024) may align with this hypothesis given the interactions between noradrenaline, arousal, glymphatic flow and sleep state.

#### 1.3.3 Plasticity, neurogenesis, and hippocampal dependent encoding of novel/salient sensory stimuli

Stimulation of noradrenergic receptors in the hippocampus alters neuronal excitability and synaptic plasticity during learning and memory encoding (Gelinas and Nguyen, 2007). Consistent with this notion, NE enhances plasticity, information transfer, and encoding during stimuli exposure for a variety of hippocampus-dependent tasks. The effects of NE receptor activation on cellular plasticity may account for NE-dependent modulation of memory in the hippocampus (Gelinas and Nguyen, 2007).

Depending on which subtype of adrenoceptor is activated, NE differently affects intrinsic membrane properties of postsynaptic neurons and synaptic plasticity (Marzo et al., 2009). For example, α1-adrenoceptor activation is mainly related to the potentiation of synaptic responses during learning and memory processes. α2-adrenoceptor activation may contribute to a higher-order information processing such as executive function.

Burst NE release transiently inhibits feedforward interneurons and either excites or inhibits subpopulations of feedback interneurons (Harley, 2007). Consistent with reduced feedforward inhibition, granule cell firing in the dentate gyrus is transiently increased. Norepinephrine selectively promotes the processing of medial perforant path spatial input through short- and long-term potentiation of cell excitability and through delayed potentiation of synaptic input. Stimuli, such as novelty, rewards, and punishment activate locus coeruleus neurons and enhance responses to medial perforant path input and engage late phase frequency-induced long-term potentiation through β-adrenoceptor activation. Behavioral studies are consistent with the mechanistic evidence for a norepinephrine role in promoting learning and memory and assisting retrieval (Harley, 2007).

Loss of NE-rich afferents from the LC ascending to the hippocampal formation has been reported to dramatically affect distinct aspects of cognitive function, in addition to reducing the proliferation of neural progenitors in the dentate gyrus (Gulino et al., 2023). Transplantation of LC-derived neuroblasts significantly ameliorated working memory performance and reinstated a normal density of proliferating progenitors after lesion of the original LC. These results indicate that LC-derived NE inputs act as positive regulators of hippocampus-dependent spatial working memory via the maintenance of normal progenitor proliferation in the dentate gyrus. Crucially, norepinephrine has been found to have opposite effects on two fundamental neurogenic niches of the adult brain as a negative regulator of adult periventricular neurogenesis and a positive regulator of neural progenitor cell (NPC) proliferation within the hippocampus (Weselek et al., 2020).

Combined destruction of the cortical noradrenergic and cholinergic innervations reduces the physiological response to monocular deprivation although lesions of either system alone are ineffective (Bear and Singer, 1986). This suggests that both cholinergic and noradrenergic transmission facilitate synaptic modifications in the striate cortex by a common circuit or mechanism.

Noradrenaline can also robustly activate dopamine D_1_ receptors in the mouse hippocampus. Noradrenaline-activated D_1_ receptor signaling was highly sensitive to the neuronal activity and experience of mice (Kobayashi et al., 2022). Chronic stress and voluntary exercise synergistically augmented noradrenaline–D_1_ receptor signaling. This augmented noradrenaline–D_1_ receptor signaling promoted the induction of hippocampal neuronal plasticity by an antidepressant drug acting on the noradrenergic system. Further work has shown that LC-innervations, rather than VTA, release a significant portion of hippocampal dopamine during memory encoding (Krohn et al., 2023).

Loss of LC neurons and noradrenergic innervation has been observed across neurodegenerative diseases including AD and PD, where it has been found to profoundly correlate with neuropathology and cognitive/behavioral deficits (Bear and Singer, 1986; McMillan et al., 2011). The relationship between LC-health, noradrenaline, and the development of neurodegenerative disease is an ongoing topic within the field (Gutiérrez et al., 2022; Leanza et al., 2018; Weinshenker, 2018).

#### 1.3.4 Summary of mechanisms and implications for AD

Overall, the above sections highlight the potential role of cholinergic and noradrenergic inputs to the phenotypes observed following GENUS, as well as offers a potential circuit explanation for the relationship between these neuromodulators and gamma oscillation dynamics across the sensory, prefrontal, and hippocampal cortex. The specific mechanism highlighted by Murdock et al. (2024) is proposed to be a result of increased noradrenergic and cholinergic signaling to relevant cortices influencing astrocytes, inhibitory neuron transmission, and other cells in the neurogliavascular unit. Other current proposed mechanisms of GENUS have focused on specific inhibitory interneuron subtypes (PV+, VIP+) and their key role in therapeutic benefit while also observing changes in glial cell populations and phenotypes, as well as vascular flow and glymphatic clearance. We would hypothesize that these changes in GABA transmission may be mediated both through direct noradrenergic and cholinergic input to these neurons and indirect communication between neuromodulators altering astrocyte behavior and their impact on GABAergic neuronal firing.

We would highlight the specific relevance of attention and novelty to AD. Attention is known to be reduced in AD, and the ability to encode new information and ability to determine novelty of stimulus also appears to be impaired (Bastin et al., 2019; Malhotra, 2019). Notably, the LC is one of the earliest sites of phosphorylated tau tangle accumulation in AD (Harley et al., 2021). Both cholinergic and noradrenergic agonists have shown some impact on Alzheimer’s pathology and cognition (Chen et al., 2022; David et al., 2022). There is significant loss in LC volume and magnetic resonance imaging (MRI) contrast in both AD and PD (Krohn et al., 2023). The functional relationship between LC-NE dynamics and mechanisms of Alzheimer’s, amyloid accumulation, and cognitive decline are discussed in Part 3.

### 1.4 Proposed falsifiable experiments and optimization of gamma-frequency stimulation protocols

Hypothesis: With the proposed circuit above, we would suggest that gamma-frequency stimulation may mirror salient and/or novel activation (based on frequency) of relevant inputs to the ascending arousal system resulting in a noradrenergic/cholinergic response to relevant cortices to promote plasticity and response to the novel stimuli. This response may decrease with age, neurodegeneration, and LC-NE dysfunction.

Falsifiable experiment: Under this framework, disruption of phasic activation of the LC or BF or blockage of neuronal, astrocyte, or vascular adrenergic receptors in the hippocampus would significantly reduce therapeutic benefits of GENUS on synaptic plasticity, vascular oscillations and glymphatic flow, and hippocampal-dependent memory in mouse models of Alzheimer’s.

Additionally, building on the hypothesis that stimulation in the gamma frequency induces a similar “novel response” in the region that recruits LC response, we would propose that delivery could be improved by interacting with the circuit not just in terms of frequency, but also in terms of function – intermittent input or burst delivery may result in a stronger response. Specifically, the hindbrain nuclei and anterior hippocampus initiate early gamma power changes within 30–180 ms from stimulus onset in a network. Fixation patterns and the duration of the first fixation, discriminates between weakly familiar and weakly novel stimuli within 320 ms of stimulus onset (Kafkas and Montaldi, 2015). This point, between weakly familiar and weakly novel responses, is the point on the continuum where the intersection between novelty and familiarity occurs; where the behavioral responses, while accurate, are less confident (Kafkas and Montaldi, 2018). Crossing this critical threshold repeatedly in separate events may improve therapeutic response if the proposed framework is accurate.

Critically, it has been shown that LC influence on the brain is uniquely reliant on activation pattern (Vazey et al., 2018). Phasic, but not tonic activation of LC efferents are involved in salience and attention signals across the brain and that the two different firing patterns uniquely modulate the integration of sensory information in the cortex (Devilbiss and Waterhouse, 2011). Tonic versus burst LC activity uniquely shifts cortical network activity (Grimm et al., 2024). Burst stimulation protocols have shown increased therapeutic effect and reduced treatment duration in Parkinson’s and major depressive disorder (MDD) (Chung et al., 2015; Horn et al., 2020).

Hypothesis: Given this information, we would propose that burst presentation of 40 Hz multi-sensory stimulation for 300–500 ms and an inter-stimulation interval (∼600–3000 ms) may produce greater activation of novelty/saliency circuit within the hind- and forebrain nuclei compared to constant 40 Hz stimulation. Some benefits may be observed by modulating gamma-theta phase offset. Significant increases in LC-NE activation using burst stimulation may allow for reduced stimulation duration required for therapeutic efficacy similar to results using theta-gamma burst stimulation in MDD (Chung et al., 2015). The implementation of burst stimulation is a potential future experiment, but negative results would not directly refute the proposed framework.

Cont.: Additionally, delivery of stimulation to or engagement of the prefrontal cortex and sensory cortex simultaneously may increase efficacy as well. That is, multi-sensory 40 Hz stimulation, delivered simultaneously with transcranial stimulation or during learning behavior of a particular cognitive task, may increase LC-NE activation and changes in phenotypes of interest. Simultaneous performance of a working memory task was found to modulate the flow of information during gamma stimulation (Mlinaric et al., 2025).

Cont.: Finally, given this circuit function, we would suggest that rather than maximizing 40 Hz response within the sensory cortex, the interaction between sensory and prefrontal gamma oscillations or frontal-posterior theta-gamma coupling may be more relevant to therapeutic benefits. Studies should focus on stimulation protocols that produce a maximum response from the LC (functional magnetic resonance imaging, heart rate variability, or pupillometry) and/or noradrenergic and cholinergic input to the circuit of interest to maximize impact on plasticity, inflammation, neurodegeneration, and cognition in the disease model of interest. We hope that future work elucidating the mechanism and optimizing the delivery of gamma stimulation will enhance its utility in the clinical applications briefly noted above and explored in greater detail in Parts 2 and 3.

## 2 The potential role of the locus coeruleus, neuromodulatory tone, and gamma oscillations in consciousness and neuropsychiatric disease

### 2.1 Introduction

In Part 1, we discussed how interfacing with gamma oscillations and a circuit for acute recording and encoding of novel sensory stimuli may have a profound effect on neurogliavascular function and network dysfunction via noradrenergic and cholinergic signaling, especially in the setting of Alzheimer’s disease (AD). While counterintuitive when only thinking about attention and encoding, Alzheimer’s research has historically indicated a prominent role for noradrenergic and cholinergic signaling in disease and symptom-modifying pharmaceutical interventions (Bastin et al., 2019; Chen et al., 2022; David et al., 2022; Malhotra, 2019). Here, we expand on this framework and speculate on a larger role for the hind-, mid-, and basal forebrain nuclei and resulting gamma oscillations in managing consciousness, awareness, and encoding in the short- and long-term through modulation of neurogliavascular interactions and inhibitory transmission to influence attention, salience detection, and novelty encoding. The locus coeruleus (LC) and midbrain nuclei receive not only sensory information but also autonomic and interoceptive input (Pace and Myers, 2024). We have discussed how the vagus nerve provides other environmental and internal stimuli such as satiety, peripheral inflammation, proprioception, and cardiovascular state to the nucleus tract solitarus, which shows extensive LC connectivity, but additional connections between the LC, amygdala, and hypothalamic-pituitary-adrenal (HPA) axis play a pivotal role in fight, flight, and freeze responses to threatening stimuli acutely and chronically along hormonal and vagal output pathways (Morris et al., 2020; Prescott and Liberles, 2022). Activation of hind-, mid-, and forebrain nuclei and downstream hypothalamic nuclei can induce significant shifts in body state through changes in autonomic and inflammatory tone in response to emotion, stress, or strongly salient stimuli (Myers et al., 2017). The default mode network (DMN), regulating internal thought and memory, is largely maintained by the precuneus and cingulate cortex; both of which show strong connectivity with the LC which acts as the switch between the DMN and central executive and salience networks responsible for external attention (Ross and Van Bockstaele, 2021). LC activity plays a key role in the micro and macrostructure of sleep function and behavior (Krohn et al., 2023). The role of gatekeeping new information being encoded while also managing basic needs and responding to stress or potential harmful stimuli may indicate a prominent role for the neuromodulator system not just in attention, but also in consciousness, sleep, managing allostatic load, and evolutionary drive.

Overall, we explore a theoretical hierarchy of nuclei and neuromodulators which may reflect the harmonic nature of information carried in network cortical oscillations. We discuss the possibility of subconscious search, attention, engagement, and encoding, observed through 4 Hz, 10 Hz, 20 Hz, and 40 Hz activity, reflecting switching of subsets of hind-, mid-, and basal forebrain nuclei from tonic to phasic activity.

Given this proposed hierarchy, we would anticipate alterations in signaling from the hind- and midbrain nuclei to play a prominent role in neuropsychiatric disorders. Altered gamma oscillations and neuromodulatory tone have been observed across a variety of such disorders including post-traumatic stress disorder (PTSD), attention deficit hyperactivity disorder (ADHD), schizophrenia, autism spectrum disorder (ASD), and major depressive disorder (MDD), each of which are discussed in depth below. We review work indicating that hind-, mid-, and forebrain nuclei and neuromodulatory tone, and therefore gamma oscillations, attention, and memory encoding, are heavily impacted in major neuropsychiatric disorders which may play a significant role in behavioral phenotypes. This hypothesis explores a role for neuromodulators beyond passive response to cortical input, but in organization of brain state, consciousness, and memory encoding. We propose that hind- and midbrain nuclei dysfunction and resultant circuit dysfunction may be significant upstream contributors to the above disorders, and that the LC-NE system may be a therapeutic target to induce plasticity, reset network circuits, and influence these disorders.

### 2.2 The LC as a critical orchestrator of consciousness and allostatic load management

As of Breton-Provencher et al. (2021), the two predominant theories of the function of the LC-NE system in consciousness were the adaptive gain theory and the network reset theory (Aston-Jones and Cohen, 2005; Bouret and Sara, 2005; Breton-Provencher et al., 2021; McEwen and Gianaros, 2011; Yu and Dayan, 2005). The adaptive gain theory proposes that phasic activity in the LC during optimal behavioral performance facilitates task-specific decision processes, with tonic activity prevailing during periods of poor performance, and a general increase in LC-NE activity increases the gain of a network indiscriminately, making targeted circuits more responsive to any stimulus (Dayan and Yu, 2006). Thus, through adaptive gain, LC-NE activity optimizes the tradeoff between exploitation and exploration behaviors. The network reset theory suggests that contexts requiring a change in behavior transiently activate LC-NE neurons. These activating contexts lead LC-NE neurons to induce widespread cortical arousal and cause a reset in network activity to enable an updating of priors. In addition to the above, recent research has also shown a prominent role for the LC-NE system in sleep architecture and glymphatic clearance discussed below.

**FIGURE 4:**
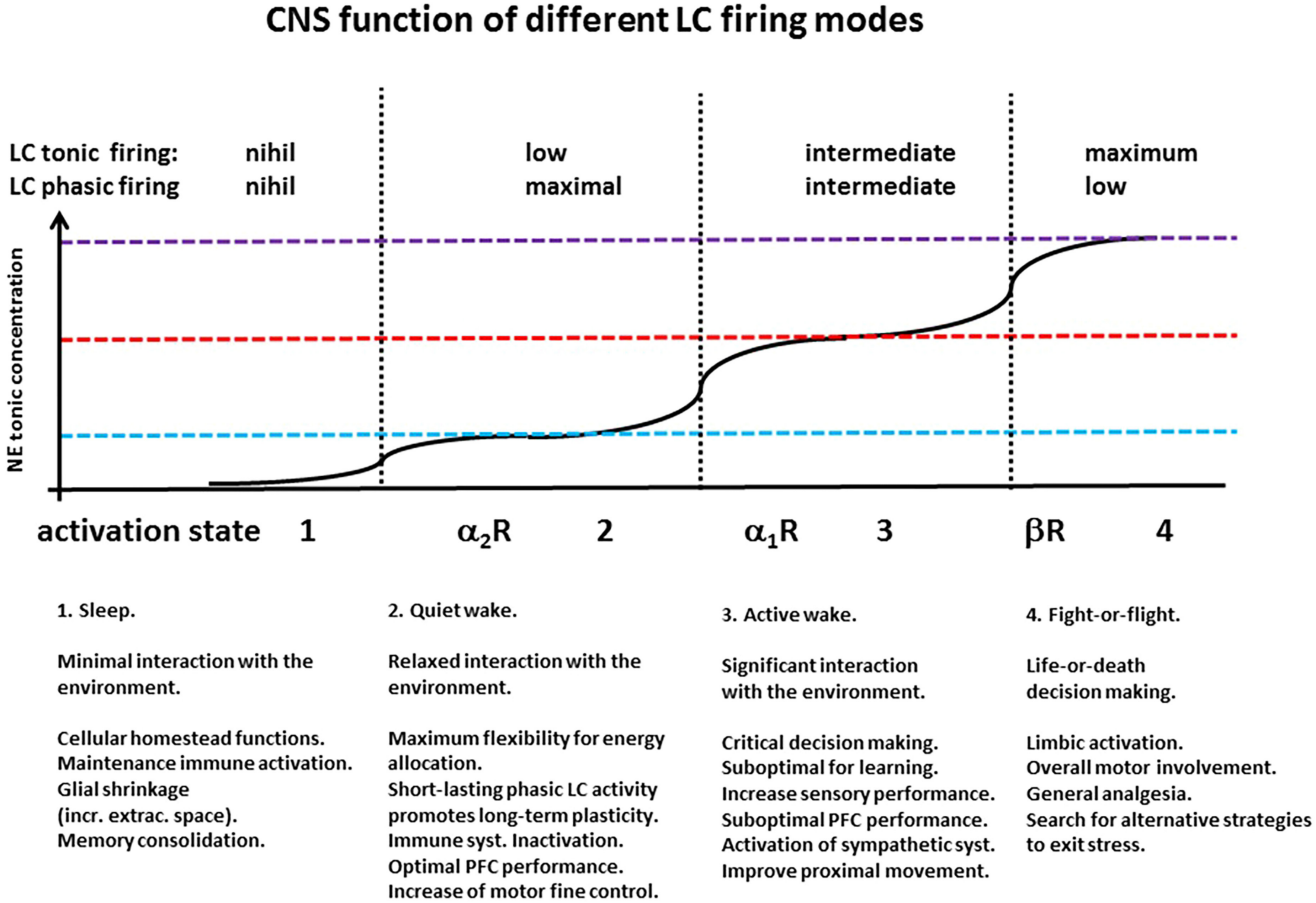
Influence of tonic and phasic LC activity. From Atzori et al. (2016), Aston-Jones and Waterhouse (2016) seminal work from Aston-Jones groups has shown the existence of a relationship between behavioral states and LC tonic and phasic firing patterns: During sleep, LC cells display low or no activity (vertical axis in arbitrary units–A.U.); during quiet wake they display modest tonic firing, and phasic responses to behavioral stimuli; in conditions of intermediate tonic release, associated with moderate stress and energy demand, LC presents its highest phasic response during biologically relevant behavioral responses; the highest level of LC tonic firing occurs in situations of arousal and fight-or-flight response, and is associated with the lowest levels of phasic LC activity.

The LC is tightly linked to sleep architecture - decreases in LC tonic and phasic activity and noradrenergic levels are linked to the transition from wakefulness to sleep, during which the LC is virtually silent during rapid eye movement (REM) sleep; similarly, increases in LC activity precede unprovoked rousing from sleep (Figure 4; Krohn et al., 2023) LC activity during non-REM sleep has been linked to memory consolidation and sleep spindle activity.

Norepinephrine-mediated slow vasomotion waves are key to driving glymphatic clearance during sleep (Hauglund et al., 2025). The dramatic shift in interstitial volume during sleep is mediated via NE-signaling, causing a reduction in astrocyte volume and allowing for increased CSF flow through tissue (Sriram et al., 2024). The LC projects to and receives input from widespread brain regions and is involved in numerous functions related to cognition such as memory formation, attention, sensory processing, novelty, and emotional memory. It is involved in autonomic functions such as blood pressure, immune function, and the sleep-wake cycle [for a review see Osorio-Forero et al. (2022)]. Additionally, it is involved in the fight or flight response by modulating heart rate, blood pressure, salivation and pupil dilation (Ross and Van Bockstaele, 2021).

Hypothesis: We propose a theoretical neuromodulatory framework in which the hind-, mid-, and basal forebrain nuclei may act as a set of neuronal frequency modulators, interacting with and influencing interneuron-neuron firing through modulation of astrocytes and the neurogliavascular unit and operating on different frequency harmonics. During wakefulness, subconscious sampling may occur around 4 Hz tonic activation or below. Initial deviation from local neuronal oscillations would be represented by spontaneous asynchronous firing to produce gamma oscillations in response to unfamiliar or unexpected stimuli (PING network). Initial attention and response may occur through cholinergic release and inhibition of relevant and non-relevant sensory neurons, respectively, altering 10 Hz oscillations and cholinergic input to prefrontal cortex for conscious attention. A secondary response would require dopaminergic input to the prefrontal and motor cortex to initiate executive function and motor planning, respectively, which is often associated with 20 Hz or beta oscillations. Temporary encoding within the entorhinal/hippocampal cortex may depend on serotonergic input, but, significantly, the long-term extraction of relevant memory engrams and learning would be represented by LC activation inducing 40 Hz or gamma oscillations through direct (NE) and indirect [acetylcholine (ACh), dopamine, and serotonin] mechanisms. This would theoretically place LC-NE activation near the top of this hierarchy to execute sustained attention, interaction, encoding, and learning.

The hierarchical nature of this evolutionary process (search, attention, planning, action, encoding, and learning as sequential filters) may be reflected in the function of large system oscillations and production of harmonics. Additionally, the proposed primary frequency of encoding-relevant cortical neuronal oscillations (40 Hz/gamma) may be a natural reflection of previously observed interaction time between interneuron-neuron units, though individual neurons fire at much higher frequencies and a high-gamma theory of information also exists (Milicevic et al., 2023). Finally, the maintenance and daily reset of this system may be influenced or orchestrated by noradrenergic LC input, glymphatic flow, amyloid clearance, and transfer of memory engram from temporary (hippocampal/entorhinal) to long-term (cingulate cortex) neurons during sleep.

### 2.3 Role of LC-NE and gamma oscillations in neuropsychiatric disorders

As outlined above, LC-NE dynamics play a role not only in arousal, attention, and encoding response to novel environmental experience, but also contribute to the cognitive, behavioral, and body response to new internal experiences, and emotional stimuli. Additionally, the LC influences wakefulness and the process of sleep, hormonal signaling, and pain, autonomic and inflammatory tone (Ross and Van Bockstaele, 2021).

Hypothesis: Overall, we would expand on previous theories related to the function of the LC-NE in vertebrates, to suggest a critical role in management of consciousness, evolutionary drive (or allostatic load), and development and learning. We would hypothesize that changes in LC-NE signaling and function, as well as downstream catecholamines, would thus play a significant role in major neuropsychiatric disorders and that this impact could be observed through altered gamma oscillations, changes in neuromodulatory tone, and impairments in attention, interaction, encoding, and recall, as well as reflected in current standard of care.

#### 2.3.1 Post-traumatic stress disorder (PTSD)

In the setting of acute or chronic traumatic exposure, particularly during development, some individuals may develop PTSD or complex PTSD, respectively. Symptoms of re-experience, avoidance of associated stimuli, negative alterations in cognition and mood, and alterations in arousal and response characterize these disorders (American Psychiatric Association, 2013). Additionally, resultant depression and anxiety disorders are significantly associated with altered oxidative stress and inflammation profiles compared to those without PTSD, highlighting the significant interaction between conscious experience, neuropsychiatric symptoms, inflammation, and oxidative stress (Kim et al., 2020).

Evidence suggests that many of the primary symptoms of PTSD, including hyperarousal and sleep dysregulation, can be viewed as a disorder of the underlying fear response of an organism. The neural circuitry underlying fear and threat-related behavior and learning in mammals has largely focused on amygdala–hippocampus–medial prefrontal cortex circuitry (Ressler et al., 2022). The anterior cingulate plays a significant role in long-term storage of emotional content and its recall (Rolls, 2019). Another extremely significant component in this fear response and resultant alterations in autonomic tone is the LC-NE system, which plays a role in long-term impairment of neuroplasticity in PTSD (Borodovitsyna et al., 2018; Giustino et al., 2019).

Several studies have highlighted the important role of stress hormones and norepinephrine during emotional memory encoding (Hruska et al., 2014). Functional changes in LC activity and output have been observed in many studies of PTSD and shown to significantly correlate with behavioral phenotypes (McCall et al., 2024; Naegeli et al., 2018). The amygdala shows significant connectivity and interaction with the LC during both acute and chronic stress responses (Zhang et al., 2021).

In terms of changes in gamma oscillations across the cortex, PTSD patients’ response to trauma scripts showed increases in high-gamma band power in visual areas (Reuveni et al., 2022). Increased frontal gamma power has also been observed in PTSD groups compared to anxiety disorder (Moon et al., 2018). Çalışkan and Stork (2019) provides a full review of PTSD and alterations in hippocampal, prefrontal, and amygdalar gamma oscillations during fear retrieval and extinction (Çalışkan and Stork, 2019).

Hypothesis: Under the proposed framework, with the LC as a central component, we would hypothesize that PTSD phenotypes may reflect inappropriate overactivation of initial fear response to similar/remembered stimuli and an inability to achieve the level of plasticity required to alter these associations. That is, the propensity of the LC to respond to and promote anterior cingulate/amygdalar activation and communication of fear/alert with the prefrontal cortex may override the ability of the LC to promote plasticity and altered association encoding within the hippocampus (fear > novelty). As a result, we would observe an increase in gamma oscillations in the prefrontal cortex, aligned with cited data (Çalışkan and Stork, 2019), activation of peripheral arousal system, amygdalar-LC activation in response to fear-associated stimuli, and reduced hippocampal noradrenergic tone indicating a decrease in encoding. Additionally, areas of long-term emotional memory storage, such as anterior cingulate cortex and basolateral amygdala, may be likely to show altered gamma activity as fear memories are recalled and activated even without environmental input.

In terms of neurostimulation, large improvements in PTSD have been seen across both biological and cognitive phenotypes using transcranial alternating current stimulation (tACS) and transcranial magnetic stimulation (TMS) targeting the posterior cingulate cortex, hippocampus, and amygdala in the gamma frequency range (Koek et al., 2019). Ketamine therapy interacts with LC receptors, influences plasticity and inflammation, and specifically has shown a significant role in modulating plasticity of amygdalar connections (Debowska et al., 2023; Fortin et al., 2023; Scheidegger et al., 2016). More standard treatment options involve serotonin or norepinephrine reuptake inhibitors (SSRI/SNRIs) and medications such as prazosin and clonidine, which operate via adrenergic signaling (Ehret, 2019). We would suggest that accompanying neuromodulation techniques with traumatic memory recall and gamma frequency sensory stimulation may promote plasticity and circuit reset through promotion of LC-NE system activation. Since improvements in depressive phenotypes have been reported in chronic unpredictable stress models after 40 Hz light stimulation via dopaminergic signaling, we would hypothesize an upstream role of the LC in activation of dopaminergic nuclei and dopaminergic release in the hippocampus (Guo et al., 2026).

#### 2.3.2 Attention-deficit (hyperactivity) disorder (AD(H)D)

Attention-deficit (hyperactivity) disorder is a developmental disorder that negatively affects several life domains. The main behavioral symptoms of the disorder, as described in the Diagnostic and Statistical Manual of Mental Disorders (DSM), are manifestations of inattention, hyperactivity, and impulsivity, which start in early childhood and persist (especially the inattentive symptoms) into adulthood in about two-thirds of diagnosed cases (Mohamed et al., 2021). Impairments in complex cognitive functions such as executive functioning and memory have also been reported in ADHD (Banaschewski et al., 2017; Fuermaier et al., 2013).

In its current framework, ADHD experiences both high variability in cognitive domains impacted and in clear diagnosis and patient populations (Pettersson et al., 2018). A frequency-based cluster analysis reported higher high-gamma power (>65 Hz) for ADHD patients over posterior sensors and lower high-gamma power for ADHD patients over frontal-central sensors (Dor-Ziderman et al., 2021). These results were shown to be stable over three measurements, unaffected by administration of methylphenidate, a common ADHD medication, and linked to cognitive accuracy and state anxiety. We propose that this pattern could reflect the overactivation of initial attention and sensory intake coupled with reduced activation of dopaminergic input and prefrontal gamma oscillations. Furthermore, the differential high-gamma activity evidenced substantial ADHD diagnostic efficacy, comparable to cognitive and emotional factors (Dor-Ziderman et al., 2021).

A dopaminergic theory of ADHD has long been hypothesized (MacDonald et al., 2024). Mouse models of ADHD have also indicated a reduction in nicotine-stimulated norepinephrine release in the hippocampus (Sterley et al., 2014). Through neuromodulatory influence over fronto-striato-cerebellar circuits, dopamine and noradrenaline play important roles in high-level executive functions often reported to be impaired in ADHD. Medications used in the treatment of ADHD (including methylphenidate, dextroamphetamine and atomoxetine) act to increase brain catecholamine levels (Del Campo et al., 2011). Increased LC neuromelanin-magnetic resonance imaging (MRI) intensity has been observed in children with ADHD and autism spectrum disorder (Al-Saoud et al., 2025). Reduced temporal complexity in eye movements, regulated by LC-NE dynamics, has also been observed in ADHD (Ueno et al., 2025).

Hypothesis: We propose that in the context of ADHD, the natural trigger for salience–cholinergic signaling to prefrontal cortex (PFC)–is present or hyperactive, but that reduced dopaminergic neuron activation or transport to the PFC results in a failure of executive function activation, stimuli interaction and discovery, and reduced probability for LC-NE input to the hippocampus to encode relevancy of (somewhat) salient stimuli. This could be due to genetic or developmental factors in certain subgroups of ADHD, but similar cognitive issues could arise in any issue impacting the ratio of cholinergic to dopaminergic inputs to the prefrontal cortex, noradrenergic input to the PFC, or upstream LC-NE activation. From this framework, we would propose that genetic predisposition or environmental stimuli alter neuromodulatory tone during development that persists into adulthood in certain subgroups of ADHD. This change in hind- and midbrain nuclei interactions and dopaminergic input to the prefrontal cortex may be observed as changes in reduced hippocampal norepinephrine release and PFC dopaminergic input, altered hippocampal and prefrontal gamma oscillations, and issues in sustained attention and encoding except in the case of highly salient or novel stimuli. Gamma stimulation of dopaminergic input sites in the prefrontal cortex or direct LC manipulation may have more pronounced cognitive benefits while gamma sensory stimulation may provide more general benefits to sustained attention and encoding during learning tasks. GENUS improved attention task performance in healthy adults, as well as attention and executive task performance in developmental age children after a single session (Attokaren et al., 2026; Lembo et al., 2026).

#### 2.3.3 Schizophrenia

Individuals with schizophrenia show diverse symptoms and deficits in multiple domains of perception and cognition (Levey et al., 2022). These include cognitive disturbances, such as attention deficits and delusional ideation; disturbances of self-awareness and agency; alterations in emotional expression; disturbed motor behavior; and sensations in the absence of external stimulation, or hallucinations. One recent model explaining these disparate presentations is the “disconnection hypothesis,” which proposes that schizophrenia disrupts signaling among brain regions, systems, or cellular circuits (Levey et al., 2022). Other studies have indicated subcortical dopaminergic function and increased inflammatory dysfunction as key factors to pathophysiology (Luvsannyam et al., 2022).

Previous studies have associated positive symptoms of schizophrenia (hallucinations, delusions, changes in speech) with hyperactivity of the NE system and negative symptoms (anhedonia, social withdrawal, and reduced speech output) with hypoactivity of the NE system (Bowie and Harvey, 2006). Together, these findings indicate a loss of differentiation in LC activation between phasic and tonic activity (Yamamoto and Hornykiewicz, 2004). Functional magnetic resonance imaging (fMRI) reveals reduced LC-DMN deactivation despite increased cognitive load in schizophrenia patients compared to controls during a working memory task (Suttkus et al., 2021).

Although increased synchronization of gamma oscillation has been reported in conjunction with symptoms of schizophrenia, patients with schizophrenia showed lower mean gamma synchronicity when compared to control subjects (Pellegrino et al., 2023). Similarly, patients with schizophrenia have been found to have deficits in auditory steady-state response upon exposure to auditory stimuli, including lower spectral power from frontal measurements in response to 40 Hz click trains. Finally, changes in LC-NE activity, measured by task-evoked pupil dilation, in response to increases in task demand are also correlated with schizophrenia symptoms. Changes in LC-NE activity when task demand increases – measured by task-evoked pupil dilation – are associated with schizophrenia symptoms (reduction in response) (Shin et al., 2011).

Mäki-Marttunen et al. (2020) propose that schizophrenia, in particular cognitive symptoms, may be explained by an abnormal interaction between genetic susceptibility and stress-initiated LC-NE dysfunction. This in turn, leads to imbalance between LC activity modes, dysfunctional regulation of brain network integration and neural gain, and deficits in cognitive functions.

Hypothesis: Overall, similar to Mäki-Marttunen et al. (2020), we would propose that schizophrenia may arise from a genetic predisposition for LC dysfunction, exacerbated by trauma, stress, or environmental input, which impairs the circuit ability to shift appropriately between tonic and phasic activity. In turn, this may reduce the ability of the cortex to appropriately discern internal from external stimuli and/or differentiate between sensation and memory recall. The potential impact of gamma frequency stimulation on cognitive deficits is unclear, but we would hypothesize that functional normalization of LC activation may provide cognitive benefits.

#### 2.3.4 Autism spectrum disorder (ASD)

Autism spectrum disorder is a neurodevelopmental disorder characterized by social communication impairments and restricted, repetitive behaviors (Beversdorf, 2020). Agents that decrease activity of the noradrenergic system have been used for anxiolytic and behavioral purposes in ASD. Early reports indicated benefits in language and social behaviors in a consecutive case series of individuals with ASD treated with β-adrenergic antagonists. α_2_ adrenergic agonists, drugs which act to presynaptically inhibit norepinephrine release, have been shown to improve hyperactivity, impulsivity, hyperarousal and social relationships in individuals with ASD. A number of researchers have demonstrated findings suggestive of increased noradrenergic activity in ASD, including increased plasma epinephrine and norepineprine; however, subsequent studies demonstrating no abnormalities in basal noradrenergic functioning have indicated that increased reactivity to clinical procedures may have led to the earlier atypical findings (Levey et al., 2022). Although resting state gamma oscillations have found conflicting results, meta-analysis indicates an increase in gamma power in individuals with ASD (Neo et al., 2023).

Multiple studies have reported that atypical visual perception in ASD, such as faster detection of visual targets but reduced perceptual integration among multiple elements, occurs with reduced gamma responses in posterior cortical regions compared to controls, as well as invariant gamma response to increases in the contextual modulation of the stimuli (i.e., increased number of homogenously aligned elements) (Milne et al., 2009; Snijders et al., 2013). Others have found that a deficit in ascending cerebellar Purkinje fibers, which provide significant regulatory input to the LC, is often observed in individuals with ASD (Hampson and Blatt, 2015). It has also been observed that febrile episodes may temporarily ameliorate autistic behaviors and improve communication, which may be a result of functional normalization of LC-NE systems (Mehler and Purpura, 2009). However, recent single-genome sequencing work has instead implicated an evolutionarily recent group of excitatory neurons responsible for communication between neocortical regions (Starr and Fraser, 2025). Others have shown amelioration of cognitive phenotypes following transcranial stimulation of prefrontal regions involved in social responsiveness (Luckhardt et al., 2025).

Hypothesis: The interaction between LC-NE activation, gamma oscillations, and ASD is not as clear as in other disorders discussed above. While the underlying mechanism seems to be similarly related to LC-NE and gamma oscillation involvement, the exact impairment remains unclear. We would hypothesize that ASD may relate to an inability, dysfunction, or reduced tendency to extract or respond to salient cues from social stimuli, especially during development, and to give them proper valence during memory encoding due to alterations in function or connectivity with cortical regions or neurons relevant to these interactions and the LC. We might expect to see reduced gamma oscillations in the hippocampus in response to social stimuli, indicating a lack of associated salience or importance of these stimuli to LC-encoding. In alignment with this hypothesis, altered gamma oscillations, reduced inhibitory input, and altered theta-gamma modulation have been observed in mouse models of ASD (Paterno et al., 2021). A reduction in LC response to social stimuli could mean a reduction in overall gamma oscillation power in sensory/relevant cortices compared to control as the individuals have reduced motivation to interact and engage and therefore a reduction in behavioral adaptation in response to social stimuli. GENUS significantly reduced deficits in social novelty in a 16p11.2 deletion mouse model (Ju et al., 2024).

#### 2.3.5 Major depressive disorder (MDD)

Major depressive disorder is characterized by physical changes such as tiredness, weight loss, and appetite loss (Cui et al., 2024). Anhedonia or emotional blunting is a classic feature of MDD and is often accompanied by lack of drive, sleep issues, and cognitive challenges. The clinical symptoms of MDD include a depressed mood, loss of interest in normally stimulating activities, changes in weight or appetite, and increased likelihood of committing suicide (Rice et al., 2019). Additionally, MDD has been found to have high comorbidity with cardiovascular disease, reduced heart rate variability associated with functional nodes of the frontal-vagal network, and other alterations in autonomic tone (Iseger et al., 2020).

**FIGURE 5:**
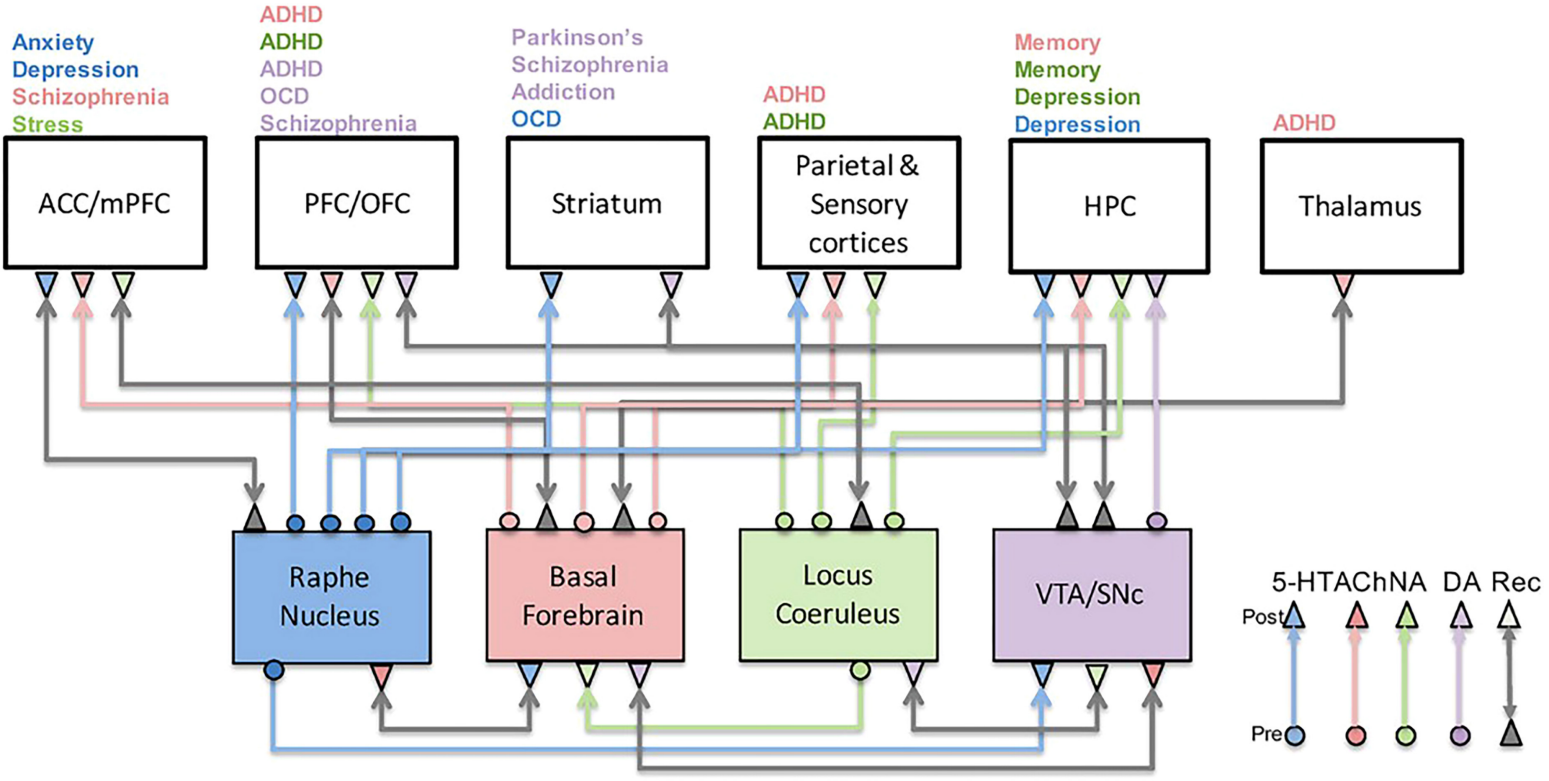
Neuromodulatory system interactions and their role in disease. From Avery and Krichmar (2017) this figure illustrates how serotonergic (blue), cholinergic (red), noradrenergic (green), and dopaminergic (purple) systems are highly connected to one another as well as cortical and subcortical structures. Their malfunction has been associated with a host of neurological and psychiatric conditions as indicated above each brain region. Gray arrows denote recurrent connections.

Overall, across a variety of major neuropsychiatric disorders discussed above, a multitude of streams of research have converged around alteration in noradrenergic signaling (and downstream neuromodulators – cholinergic basal forebrain, dopaminergic neurons in ventral tegmental area, serotonergic neurons in raphe nucleus) which significantly impact conscious experience and the ability or propensity to interpret, encode, and respond appropriately to external stimuli (Figure 5). These functional deficits have also been observed with alterations in gamma oscillations during stimuli or task presentation.

Many of these disorders reflect acute response interpretation and the altered neuromodulatory signaling or thresholds for activation altering biological and conscious response.

Hypothesis: We propose that acute depression is a short-term (weeks - months) downregulation of serotonergic tone and that MDD involves a long-term (years – decades) reduction in general noradrenergic input due to inability to meet allostatic needs. Mechanistically, MDD would be a reduced likelihood for LC-NE activation for learning behavior and regulating allostatic load. This downregulation of the LC-HPC-PFC pathway could occur for a variety of reasons including chemical, environmental, genetic, or behavioral origins, but the result is a decreased ability to achieve salient, emotional or executive response to stimuli, reduced novelty seeking, and decreased likelihood to encode new information or emotional content associated with it. Consistent with our hypotheses is previous research indicating altered gamma oscillations, across the prefrontal, hippocampus, and cingulate cortex in major depression, which has prompted its proposed use as a biomarker (Fitzgerald and Watson, 2018). Additionally, the LC has been shown to have a significant role in social-defeat induced inflammation (Finnell et al., 2019). The historical use of monoamine oxidase inhibitors and selective serotonin and norepinephrine reuptake inhibitors, as well as the rise of transcranial stimulation techniques to treat MDD, also aligns with this hypothesis (Cui et al., 2024).

Acute instances of depression may be treated with serotonin reuptake inhibitors, but major depressive disorder (MDD) may be more likely to require LC-specific or noradrenergic transport intervention. Chronic 40 Hz sensory stimulation may have a positive impact on MDD symptoms and cognitive phenotypes. 40 Hz violet light stimulation found improvements in depression rating scales in participants with MDD (Noda et al., 2025).

### 2.4 Future indications, proposed experiments, and implications for AD

Overall, in the context of the neuropsychiatric disorders discussed above, the LC is situated as a hub of significant influence on brain arousal and consciousness, a functional conductor of sleep dynamics and clearance, and a source of immense influence on motivation, evolutionary drive, and basic function of memory encoding for relevant stimuli both directly through NE input or indirectly through downstream activation of other relevant nuclei and circuits. For this reason, gamma oscillation modulation techniques, invasive or otherwise, are well-positioned for symptom alleviation and disorder modification when considering the neuromodulatory dysfunction of the disorder and its relevant cortical regions.

∼6.1% of Americans are prescribed a stimulant based on dopaminergic and noradrenergic reuptake inhibition, while the prevalence of antidepressant use, many of which operate on serotonergic, noradrenergic, or dopaminergic reuptake inhibition, is greater than 22% (Kay et al., 2025; Sanchez-Ruiz et al., 2023). Additionally, recent work has indicated a significant role of LC-NE dynamics in the presentation of neuropsychiatric symptoms early in Alzheimer’s disease (Korukonda and Weinshenker, 2026). Beyond their potential as early indicators, many of these neuropsychiatric diseases or behaviors predispose or increase risk of development of AD (Hedges et al., 2024).

Falsifiable experiments: Initial results have indicated a positive impact of GENUS on attention, depression, chronic stress, and social behavior in preclinical models, while clinical trials are in progress. Noradrenergic interventions for many of these disorders currently exist as frontline treatment. We would hypothesize that improved models of neuropsychiatric disorders with a focus on LC-NE dynamics and connectivity may more accurately reflect clinical behavioral phenotypes. Modifications of dopamine β-hydroxylase, which controls the synthesis of norepinephrine, have been used in preclinical studies of ADHD, anxiety, depression, and chronic stress, but chronic functional disruption of LC-NE activity, with consideration for early life stress, may more appropriately recapitulate disease phenotypes compared to other mouse models.

## 3 A potential role for the locus coeruleus in managing brain and body aging and its implications in Alzheimer’s disease

### 3.1 Introduction

So far, we have reviewed a large set of literature highlighting the functional role of the locus coeruleus and norepinephrine (LC-NE) system in attention, salience, and memory encoding through interaction with the neurogliavascular unit, inhibitory transmission, and gamma oscillations, as well as its possible roles in influencing several major neuropsychiatric disorders and managing consciousness, arousal, and sleep. This section will explore an evolutionary theory on the functional hierarchy of the LC-NE system and its downstream signaling pathways, as well as its potential impact on biological aging and Alzheimer’s disease (AD).

The evolutionary goal of any organism and its nervous system is to find new and improved ways to sense and interact with its environment for pro-survival information or resources without expending extraneous energy (Mobbs et al., 2015). Higher cognition correlates with increased ability to determine what information is relevant for a given stimulus and the ability to store and apply that information to novel or unrelated situations. While the ways in which we are able to acquire information and the ways our neocortex can store and recall memory have grown increasingly complex, the ultimate evolutionary decisions come down to “What is worth attention, What is worth spending energy on interaction, and What information is most important to store – short- or long-term?” We have outlined how the LC-NE system plays a significant role in making and enacting these decisions through its influence on cortical networks, plasticity, and conscious state. It is likely important that these decision-making processes come down to (relatively) simple neuronal function because they are continuous calculations carried out in all living vertebrates. In the absence of higher cognition, the ability to alter this process in response to new information may not be especially prominent or useful, whereas higher cognition organisms may be more likely to alter the basic decision-making process involved in response to new information (Botero, 2026).

It can be noted that plasticity, encoding, consciousness, and the decision-making processes made in the context of the above questions all change drastically throughout, and influence, development and aging in vertebrates (Navakkode and Kennedy, 2024). Additionally, the environmental fulfillment we have, the amount of novel engagement and socialization, and exposure to bacterial, viral, physical, and emotional stressors all play significant roles in how we develop and age. The exact mechanism of these influences has not always been clear, but it is thought that these various stimuli may have an impact on organism-wide plasticity, myelination, inflammation, resting autonomic tone, cortical oscillations, development, and cognition (Thayer et al., 2021).

We would propose a working theory that the aging process in vertebrates is an orchestrated process influenced by LC-NE dynamics. That is, there is generally a set “lifespan” plan, that for most organisms involves a period of high neuronal and cellular plasticity during development (particularly long for humans) heavily influenced by environmental experience, a shift into adulthood and reproductive stage, followed by late-age management of biological processes under increased oncogenic risk and mechanical wear until expiration. This process may mostly be stored in (epi)genetic code; however, to allow for deviations, fluctuations, and environmental input to this process, it must be systemically influenced by a circuit that is able to modulate inflammatory tone, plasticity, and behavior to evolutionarily-relevant stimuli and perceived allostatic load in both the short and long-term. The role of this circuit, level of activity, and responsiveness of this system to new information may correlate with increased LC connectivity, higher order cognition, and increased lifespan “complexity.” We see this reflected in the difference in LC-NE structure in mice versus rats and in the number of LC neurons across species (Gasparini et al., 2021; Wang et al., 2022).

Finally, given the importance of this system, we would propose that a significant component of dementia and AD may be a result of NE insufficiency or LC-NE system dysfunction. While presymptomatic amyloid accumulation is its own process, AD would then be described as depletion or failure of the LC itself, supported with research indicating it as the initial site of tau accumulation (Harley et al., 2021). Many factors that we consider impactful in terms of organism functional age are found to be correlated with incidence of AD – socialization, physical, emotional, oxidative, and inflammatory stress, novelty, cardiovascular health, and diet all influence LC-NE health because the goal of the system is to monitor and manipulate response to these allostatic signals over time (Evans et al., 2022). Negative evolutionary/organism stressors are quantified, encoded, and provided as input to the LC to attempt to adjust accordingly. The more resources spent without resolution and the less “exercise” it receives in terms of positive mood, social interactions, and novelty, the more likely it may be to fail throughout an extended aging process. The rest of this article will attempt to describe a hypothetical framework for the process of aging and development of AD in the context of LC-NE function.

### 3.2 A brief view of biological aging

“At the biological level, aging results from the impact of the accumulation of a wide variety of molecular and cellular damage over time. This leads to a gradual decrease in physical and mental capacity, a growing risk of disease and ultimately death. These changes are neither linear nor consistent, and they are only loosely associated with a person’s age in years. The diversity seen in older age is not random. Beyond biological changes, aging is often associated with other life transitions such as retirement, relocation to more appropriate housing, and the death of friends and partners” – World Health Organization.

At a mechanistic level, aging is characterized by multifactorial pathophysiological alterations involving genomic instability, telomere attrition, epigenetic modifications, loss of proteostasis, impaired autophagy, mitochondrial dysfunction, cellular senescence, stem cell exhaustion, deregulated nutrient sensing, and altered intercellular communication (Guo et al., 2022). These interconnected processes contribute to the progressive functional decline of multiple organ systems and underlie the increased incidence of age-associated pathologies such as neurodegenerative, cardiovascular, metabolic, musculoskeletal, and immune disorders. Although advancements in medicine have markedly extended human lifespan, the concomitant rise in chronic, age-related diseases has emerged as a principal cause of morbidity, disability, and mortality in the aging population (Guo et al., 2022).

Contemporary research efforts focus on elucidating the molecular and cellular mechanisms that govern aging, with the goal of identifying therapeutic interventions capable of promoting both quality of life and longevity. Promising strategies include caloric restriction, microbiota modulation, and targeted nutritional interventions, alongside clinical approaches such as senescent cell clearance, stem cell therapy, antioxidant and anti-inflammatory treatments, and hormone replacement therapy (Guo et al., 2022). However, a deeper understanding of the molecular determinants and systemic regulation of aging may be necessary for developing more effective interventions to delay or mitigate aging-related pathologies and enhance overall organismal resilience.

Aging in the central nervous system is tied to specific structural changes, including neuronal shrinkage, degeneration of dendrites, demyelination, immune activation via microglia, reduction in metabolic rate, small vessel disease, and incidence of white matter lesions (Blinkouskaya et al., 2021). As a locus of neuroendocrine control, the hypothalamus drives several of these mechanisms of physiological degradation. Specific neuronal subsets including somatostatin (SST), vasoactive intestinal polypeptide (VIP), and gonadotropin-releasing hormone (GnRH) are thought to be responsible for metabolic, hormonal, circadian, and reproductive functional deficits, as a result of dysregulated nutrient sensing, altered intercellular communication, exhaustion of stem cells, proteostatic loss, and epigenetic changes in these cell populations (Kim and Choe, 2019).

### 3.3 Complications with current structures of aging

Current conventional descriptions of aging treat complex organisms as an assortment of independent local cellular interactions that serve to govern the functional ages of specific cell populations. However, such conventions understate the complex interactions between basic biological systems and their cellular environment, as well as the drastic changes observed across different systems in response to significant life changes, stress and trauma, and (hormone-driven) developmental shifts that are reflected in biological age and organism function.

Given the structural and functional diversities of individual tissues and their local environments, the onset and progression of age-related changes is remarkably consistent over the trajectory of natural aging, outside of tissue-specific disease contexts. We hypothesize that without a strong, unifying regulatory force, lack of coordination of aging across tissues would result in wider differences of functional age throughout the body. We would hypothesize that, on a systemic level, organisms require a central regulator to normalize the impact of exogenous stimuli and environmental experiences to produce a uniform trajectory of biological age.

We see this coordination over time as multiple systems transition from high turnover rates, pluripotency, and plasticity to more maintenance states after the development period closes and during extended aging (Gorelov and Hochedlinger, 2024). However, a large amount of research has indicated that the timing and trajectory of development period closing is heavily influenced by experience and salient stimuli (Lathe and St Clair, 2023).

If instead, we look at age from an evolutionary perspective, the goal of an organism is to develop, procreate, promote offspring survival, and eventually expire to prevent resource restrictions (Reichard, 2017). Late-aging involves increased management of oncogenic risk and mechanical tissue damage, and in a few cases such as humans, there is a post-reproductive or post-menopausal stage. Every evolutionary drive is aimed at contributing to this common pathway in some way. We would propose that as organisms developed sophisticated nervous systems to manage increasingly complex tissue interactions and stimuli, a primary role of this central controller would be to influence the increasingly advanced mechanisms that govern the transition from development and high neuronal and cellular plasticity to procreation, and then to age-related decline.

### 3.4 Justification for a neural basis of aging

Aging is complex and involves multi-system organization and coordination. The idea that the complicated process that is lifespan is governed primarily by local biochemical reactions and (epi)genetic expression without guiding input from the central nervous system is inconsistent with reported data (Lathe and St Clair, 2023). The interconnected system that influences cardiac, systemic metabolic, inflammatory, and pluripotent tone of any local tissue environment is coordinated by neural circuits and impacts cellular function, metabolism, stress, and genetic and epigenetic expression. While local stress and metabolism play a major role in the functional outcome of age, we would propose that the overall coordination guiding an organism through stages of development, procreation, and late aging is likely to be heavily influenced by neuronal systems, central planning, and organism experience, environment, and response.

### 3.5 The potential role of LC-NE in influencing and managing biological age

Important factors we have already outlined as relevant to aging include inflammatory tone, pluripotency and plasticity, metabolism, sleep structure, and cognitive ability. Other major phenotypes of aging include changes in mitochondrial energy dynamics, alterations in genetic telomeres, senescence, and reduced bone marrow function/adaptability (Sisk and Foster, 2004). Importantly, under the framework proposed, the LC-NE system would need to be able to influence these factors in the central nervous system (CNS) and periphery. In previous sections, we have discussed how the LC-NE system is able to modulate system behavior both acutely and by setting chronic tone.

In support of the LC-NE system’s potential role in coordinating aging, we can consider reported structural and functional alterations with age. Although the main rostro-caudal direction of LC is consistent across age groups, its spatial features varied with increasing age, emotional memory, and emotion regulation (Gao et al., 2022). Additionally, participants with higher-than-normal Hospital Anxiety and Depression Scale (HADS) ratings exhibited alterations in LC gradient, manifested as greater asymmetry between bilateral LC.

In addition to its influence on closing the development period in rats, the LC develops unique sex differences between males and females in puberty (Bangasser et al., 2016; Veréb et al., 2023). The greater dendritic extension and complexity seen in females predicts a higher probability of communication with diverse afferents that terminate in the peri-LC. This may be a structural basis for heightened arousal in females, an effect which may, in part, account for the sex bias in incidence of stress-related psychiatric disorders (Bangasser et al., 2011). Activation of noradrenergic neurons also influences reproductive cycle and neuroendocrine secretion, and the LC is known to play a role in reproductive senescence (Carrasco and Breen, 2025; Nicola et al., 2016). We would also hypothesize that the sexual dimorphism in structural and functional connectivity of the LC-NE may contribute to the differences in AD and Parkinson’s disease (PD) incidence rate between sexes (Pike, 2017).

In terms of diet and metabolic fulfillment, NE transporter availability has been found to be reduced in the thalamus and insular cortex in obese individuals, indicating a prominent role of the LC-NE system in maladaptive eating disorders and long-term impact of diet (Bresch et al., 2017; Li et al., 2014). Mutations in noradrenaline transporter gene SLC6A2 have been correlated with anxious arousal, PTSD, and metabolic syndrome (Lopes et al., 2016). The nucleus tract of solitarus and orexinergic neurons in the hypothalamus exhibit bidirectional innervation with the LC (Lemche et al., 2016). Generally, these results indicate a role for the LC-NE in both acute and cumulative generative measurements of allostatic fulfillment in diet, aligning with the impact of nutrition on aging and development.

Due to its exposed location next to the 4th ventricle, the LC has a relatively high vulnerability to inflammatory molecules and toxins (Evans et al., 2022; Matchett et al., 2021). Moreover, LC axons are partially myelinated and less cost-efficient, resulting in higher energy consumption, which in turn leads to higher levels of reactive oxygen species (ROS) (Lushchak et al., 2021). This predisposition to environmental toxicity sensitivity may play an intentional role in setting system-wide inflammatory tone.

Reduced leukocyte telomere length, a marker of immunological aging, has repeatedly been shown to be associated with stress response and central and peripheral (nor)epinephrine (Eitan et al., 2014). Chronic psychosocial stress through neuroendocrine mediators, specifically LC-NE activation, may drive aging through influence on cellular metabolic activity, DNA damage, telomere length, cellular senescence, and inflammatory response patterns (Polsky et al., 2022). Perceived stress and telomere length have been found to be significantly correlated, though the exact cellular mechanism remains unknown (Lin and Epel, 2022). Noradrenaline significantly impacts mitochondrial size and turnover, which could mirror the changes observed in energy dynamics throughout aging (Krajcová et al., 2022). LC and other central nervous modulators have been found to have direct connection to bone marrow tissue, allowing for modulation of blood cell production throughout lifespan (Wee et al., 2019).

During normal aging, many of the cognitive functions that exhibit decline are supported by the LC-NE, including verbal intelligence, response inhibition, memory, and emotional memory (Krohn et al., 2023).

One potential hypothesis could posit that the LC is simply the cognitive influence of the brain on aging. However, this would diminish the vast amount of information delivered to the LC of vertebrates in terms of metabolism, peripheral inflammation and infection, oxidative stress, and other metrics transmitted by the vagus nerve (Pace and Myers, 2024; Prescott and Liberles, 2022).

**FIGURE 6:**
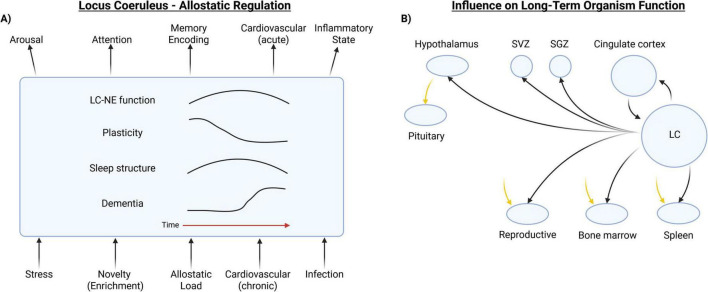
Systemic input and output of LC-NE and proposed changes over lifespan. Ways in which LC-NE influences long-term health and body-state. **(A)** Summary of some inputs (bottom) and LC-NE system outputs (top), as well as internal representation of LC-NE influence on plasticity, sleep, and development of dementia over organism lifespan. **(B)** Systemic influence of LC on long-term memory, rate of neurogenesis, blood and immune cell production and modulation directly and through hypothalamic input throughout organism aging. SVZ, subventricular zone; SGZ, subgranular zone; yellow arrows, indicate hypothalamic input or systemic influence.

Hypothesis: Instead, we would propose that the LC-NE system may play a critical upstream role in the management of biological age and the closing of the developmental period, the impact of cognitive experience on age and autonomic function, and the impact of environmental and body experience on cognition through its influence on plasticity, pluripotency, inflammation, neurogliavascular unit function, and consciousness in response to input it receives from higher cortical circuitry and the vagus nerve. Evolutionarily, this would place the LC-NE system as a core component of memory, management of lifetime stress or allostatic load, and functional biological age in vertebrates (Figure 6).

### 3.6 The potential role of LC-NE in Alzheimer’s and dementia

The locus coeruleus is the initial site of Alzheimer’s disease neuropathology, with hyperphosphorylated tau appearing in early adulthood followed by neurodegeneration in dementia (Harley et al., 2021). Locus coeruleus dysfunction contributes to Alzheimer’s pathobiology in experimental models, which can be rescued by increasing norepinephrine transmission. LC-NE dynamics contribute significantly to neuropsychiatric symptoms and sleep disruption that occurs early in AD (Korukonda and Weinshenker, 2026). Trials using a norepinephrine transporter inhibitor in subjects with mild cognitive impairment showed significant impact on cognition (Levey et al., 2022).

**FIGURE 7:**
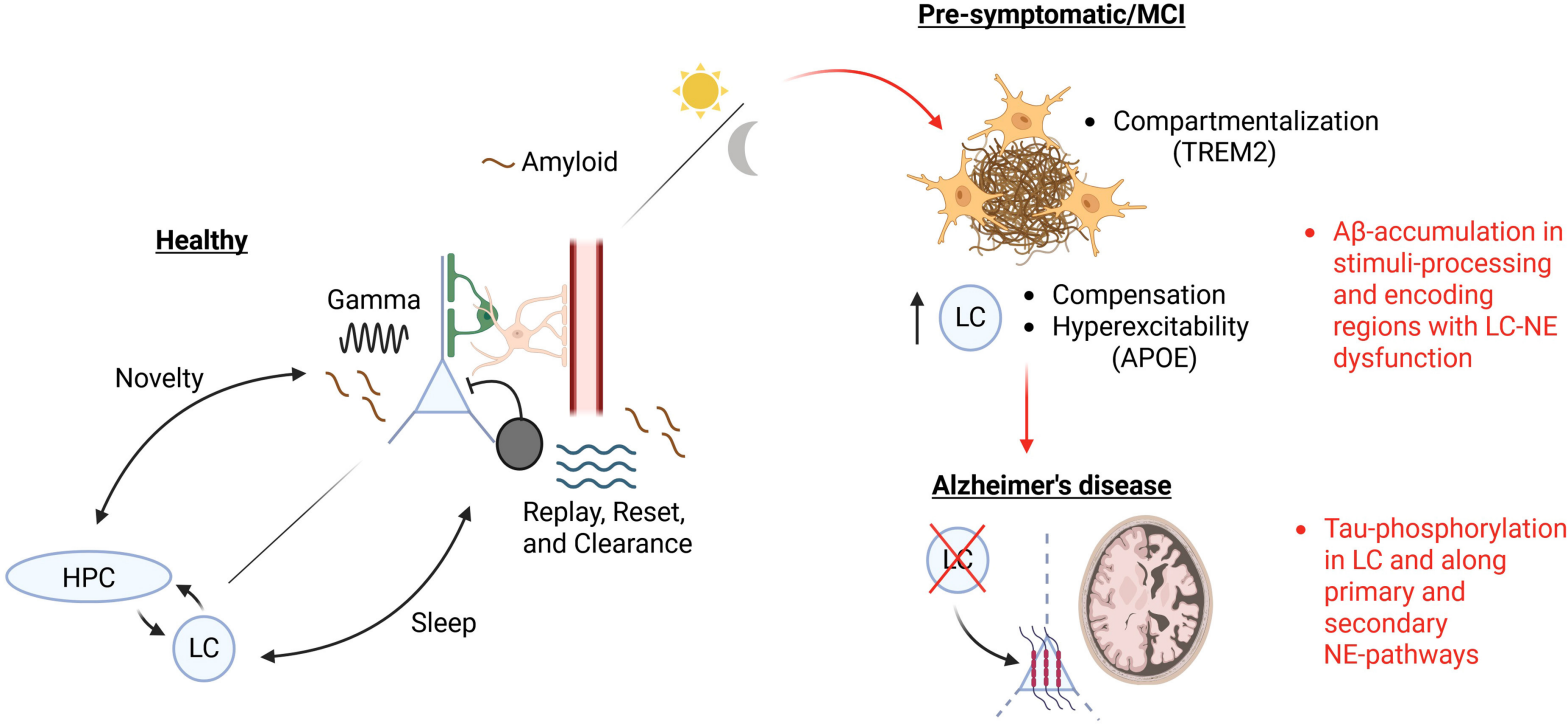
Theoretical mechanism of AD development. Amyloid protein (brown) processing plays a role in normal synaptic function and memory encoding, potentially mediated by LC-NE dynamics, gamma oscillations, and sleep (left). This process influences multiple components of the neurogliavascular unit including neurons (black, light blue), astrocytes (tan), and oligodendrocytes (green). Either encoding during wakefulness or clearance during sleep can be disrupted by a variety of factors, but the onset of mild-cognitive impairment may correlate with the compartmentalization process mediated by microglia (orange) and compensation resulting from LC hyperactivity (right). We would then propose that the onset of AD occurs when a threshold of LC damage is passed, creating a negative, progressive feedback loop involving tau phosphorylation and neurodegeneration.

Although an exhaustive review of Alzheimer’s theory is beyond the scope of this work, we expand our hypothesized role of the LC-NE system as a central manager of consciousness, evolutionary drive, and biological age, to describe a role for LC-NE insufficiency in amyloid accumulation and AD associated with exhaustion of the LC nuclei itself (Figure 7).

#### 3.6.1 LC-NE and amyloid processing

Hypothesis: In this model, we would propose that amyloid processing, a result of normal neuronal function, may occur in response to the incidence of gamma oscillations or successful LC-NE network activation during consciousness. Although this protein may merely be a metabolic byproduct, in certain forms, it may serve to mark neurons it remains in for reactivation during sleep, involvement in sleep oscillatory dynamics, and increased glymphatic clearance as a potential temporary “amyloid engram.” During sleep, LC-NE activation would then participate in reactivation of memory engrams, transfer of information to long-term memory storage, and glymphatic clearance of amyloid in relevant regions to reset encoding neurons and promote input pathways involved using previously discussed biological mechanisms.

Norepinephrine dynamics have been shown to be critical to clearance and glymphatic flow during sleep, while reduced NE in LC projection regions is associated with plaque accumulation in primate models (Duffy et al., 2019; Hauglund et al., 2025). NE stimulation of microglia reduced inflammation and increased Aβ-phagocytosis, while induced LC degeneration resulted in increased plaque accumulation and reduced microglial recruitment to plaques (Heneka et al., 2010). Aβ has been found to have a neuromodulatory and neuroprotective role in normal neuron function and to exhibit a bidirectional relationship with catecholaminergic and cholinergic signaling (Lanni et al., 2019). It is important to highlight that many decades of research have focused on amyloid deposition as a central event to AD development that then drives tau pathology, though clinical outcomes for immunotherapies have been mixed (Karran and De Strooper, 2022). No portion of the presented hypothesis challenges this view, but would instead attempt to describe LC-NE function as a critical factor in Aβ processing and clearance, as well as an upstream modulator of cognitive phenotypes and source for tau accumulation.

Various issues could arise along this proposed pathway. Lack of sleep or network function during sleep has been shown to have a significant impact on memory encoding and consciousness (Khan and Al-Jahdali, 2023). In general, LC-NE activation and function decreases with age (Budygin et al., 2025; Hämmerer et al., 2018). Cardiovascular or glymphatic dysfunction could impair the ability of the system to respond to LC-NE activation or successfully clear amyloid using the glymphatic system. Infection, inflammation, or mechanical trauma within brain tissue would also stress this system. Finally, alterations in neuromodulatory tone or network and circuit organization could result in an inability to properly activate the LC-NE for a particular region or stimulus.

#### 3.6.2 LC-NE dysfunction and onset of AD

Amyloid accumulation without glymphatic clearance causes changes in both local neurogliavascular signaling and LC response to this condition (Giorgi et al., 2020). The propensity of amyloid cleavage into different isomers may be a response to differential encoding-circuit activation or impaired glymphatic amyloid clearance. Early compensation mechanisms have been shown to include packaging amyloid into plaques by local glia to mitigate impact on circuit function (influenced by TREM2 signaling in microglia), as well as hyperactivation of LC-NE systems [impacted by apolipoprotein-4 (APOE4), observed as network hyperexcitability] (Lin et al., 2024; Nuriel et al., 2017). The initial amyloid accumulation period without cognitive dysfunction may reflect this compensation and compartmentalization process. We would propose that mild cognitive impairment may correlate with slowly increasing amyloid accumulation and, potentially, with increased LC-NE burden. However, we note that further experimental evidence is required to show whether insufficient LC input is a direct cause of amyloid deposition in human patients, as well as its significance in comparison to other modulators of Aβ processing.

Hypothesis: Alzheimer’s disease, which is associated with neuronal death and tau tangle accumulation within the locus coeruleus, could be described as exhaustion of LC nuclei in the face of failed network activation and amyloid clearance, correlating with lifetime accumulation of LC burden. Once sufficient pathology has accumulated, tau would then propagate upward along LC-NE projections. Tau accumulation has been shown to result from certain NE metabolites and propagate along ascending axons to downstream regions such as the BF (Gilvesy et al., 2022; Kang et al., 2020). While in certain situations, we may see amyloid plaque accumulation on its own disrupt acute/short-term cognitive function, early LC-NE insufficiency or development of AD may be likely to be observed in cognitive functions involving long-term encoding of memory (due to disruption of memory engram formation and subsequent reactivation during sleep), learning, and recall. Later changes in attention, executive function, and encoding could occur as a result of decreased LC-NE input (depletion or dysfunctional circuit activation) to or depletion of the basal forebrain, dopaminergic nuclei, and serotonergic nuclei, respectively.

#### 3.6.3 LC-NE and apolipoprotein E4 (APOE4)

Apolipoprotein E4 is the largest genetic risk factor for the development of AD. Recently, it has been shown that APOE4 selectively binds to the vesicular monoamine transporter 2 (VMAT2) and inhibits neurotransmitter uptake (Kang et al., 2021). The exclusion of norepinephrine (NE) from synaptic vesicles leads to its oxidation into toxic metabolite, which subsequently activates cleavage of tau and triggers LC neurodegeneration. APOE expression was found to correlate with neuromelanin content in the LC in postmortem tissue (Mulvey et al., 2025). We would hypothesize that a component of APOE4 influence on AD development may be due to its impairment of norepinephrine production and transport, and its role in early LC depletion and neurodegeneration.

### 3.7 A note toward multiple sclerosis (MS), chronic pain, and autoimmune conditions

Recent research has indicated a central role for Epstein-Barr virus in the development of multiple sclerosis (Bjornevik et al., 2023). Stress related disorders, depression, and anxiety have all been shown to significantly correlate with later development of autoimmune disorders (Andersson et al., 2015; Siegmann et al., 2018; Song et al., 2018). The development of chronic pain is often associated with trauma (Beard and Aldington, 2012).

Hypothesis: We would also propose that the influence of the LC-NE system on inflammation and brain-body interactions may play a common role in these conditions and others related to autoimmunity. That is, in MS, it may be that viral infection disrupts signaling between local regions and the information provided to the LC or LC immune response, resulting in delayed-onset activation of autoimmunity which may sporadically recur during similar vagal-mediated immune responses. LC activation has been shown to have anti-inflammatory and neuroprotective benefits in a mouse model of MS (Torrillas-de la Cal et al., 2023). Additionally, some instances of autoimmunity and chronic pain may be due to the interactions between those conscious experiences (chronic or acute), reactivation of those memory engrams, and the autonomic/vagal/inflammatory/bodily response (conscious or subconscious) to those experiences. In fact, noradrenergic neurons in the LC have already been shown to play a significant role in the development of neuropathic pain (Brightwell and Taylor, 2009; Taylor and Westlund, 2017).

### 3.8 LC-NE interventions to promote cognitive longevity

Overall, we have proposed that the primary function of the LC is not just in managing consciousness, awareness, and attention acutely, function which is reflected across neuropsychiatric disorders, but operates as a central location for memory, evolutionary drive/fulfillment, and biological age through its influence on inflammation, plasticity and pluripotency, neuroendocrine and cardiovascular system function, and cognition. Human data continues to show a role for noradrenergic and dopaminergic input from the LC correlating with memory decline in aging and AD (Dahl et al., 2023). Additionally, we would then propose that amyloid accumulation may correlate with LC-NE insufficiency and AD with depletion of the LC and its projections.

Hypothesis: Factors that reduce risk of AD, such as exercise, consistent sleep, socialization, and encountering novel experiences may serve to flex this circuit connecting allostatic load, novelty, and learning. Complementarily, reducing stress, oxidative damage, viral load, and inflammation may also increase LC-NE longevity.

Falsifiable experiments: Treatments focused on gamma oscillation stimulation, NE transport, reception, and reuptake, or direct interaction with the LC, particularly the application of LC-programmed stem cells, may provide disease-modifying benefits across dementia patient populations (Tao et al., 2024). Noradrenaline application alone without concern for functional or structural resolution is likely to have low impact. However, while LC-based interventions are likely to improve cognitive phenotypes and prevent neurodegeneration, restoration of LC-NE input and glymphatic flow may not remove densely packed amyloid plaques. Similar to ongoing trials of dopaminergic replenishment with stem cell application in PD, we would hypothesize similar or greater benefits in AD with restoration of LC function associating with cognitive improvement similar to results observed using LC-derived neuroblasts in rat models (Gálvez-Márquez et al., 2026; Gulino et al., 2023; Sawamoto et al., 2025).

## Data Availability

The original contributions presented in this study are included in this article/supplementary material, further inquiries can be directed to the corresponding author.
